# A new KSRP-binding compound suppresses distant metastasis of colorectal cancer by targeting the oncogenic KITENIN complex

**DOI:** 10.1186/s12943-021-01368-w

**Published:** 2021-05-26

**Authors:** Jeong A Bae, Woo Kyun Bae, Sung Jin Kim, Yoo-Seung Ko, Keon Young Kim, So-Yeon Park, Young Hyun Yu, Eun Ae Kim, Ik Joo Chung, Hangun Kim, Hyung-Ho Ha, Kyung Keun Kim

**Affiliations:** 1 Department of Pharmacology, Chonnam National University Medical School, Baekseoro 160, Dong-Ku, Gwangju, 61469 South Korea; 2 Department of Hematology-Oncology, Chonnam National University Medical School, Baekseoro 160, Dong-Ku, Gwangju, 61469 South Korea; 3 Immunotherapy Innovation Center, Chonnam National University Medical School and Hwasun Hospital, Hwasun, South Korea; 4 College of Pharmacy, Sunchon National University, Jungangro 225, Sunchon, 57922 South Korea; 5 College of Pharmacy, Chosun University, Gwangju, South Korea

**Keywords:** Colorectal cancer, KITENIN complex, KSRP, Metastasis, microRNA

## Abstract

**Background:**

Distant metastasis is the major cause of death in patients with colorectal cancer (CRC). Previously, we identified KITENIN as a metastasis-enhancing gene and suggested that the oncogenic KITENIN complex is involved in metastatic dissemination of KITENIN-overexpressing CRC cells. Here, we attempted to find substances targeting the KITENIN complex and test their ability to suppress distant metastasis of CRC.

**Methods:**

We screened a small-molecule compound library to find candidate substances suppressing the KITENIN complex in CRC cells. We selected a candidate compound and examined its effects on the KITENIN complex and distant metastasis through in vitro assays, a molecular docking model, and in vivo tumor models.

**Results:**

Among several compounds, we identified DKC1125 (Disintegrator of KITENIN Complex #1125) as the best candidate. DKC1125 specifically suppressed KITENIN gain of function. After binding KH-type splicing regulatory protein (KSRP), DKC1125 degraded KITENIN and Dvl2 by recruiting RACK1 and miRNA-124, leading to the disintegration of the functional KITENIN–KSRP–RACK1–Dvl2 complex. A computer docking model suggested that DKC1125 specifically interacted with the binding pocket of the fourth KH-domain of KSRP. KITENIN-overexpressing CRC cells deregulated certain microRNAs and were resistant to 5-fluorouracil, oxaliplatin, and cetuximab. DKC1125 restored sensitivity to these drugs by normalizing expression of the deregulated microRNAs, including miRNA-124. DKC1125 effectively suppressed colorectal liver metastasis in a mouse model. Interestingly, the combination of DKC1125 with 5-fluorouracil suppressed metastasis more effectively than either drug alone.

**Conclusion:**

DKC1125 targets the KITENIN complex and could therefore be used as a novel therapeutic to suppress liver metastasis in CRC expressing high levels of KITENIN.

**Supplementary Information:**

The online version contains supplementary material available at 10.1186/s12943-021-01368-w.

## Background

The overall survival of patients with colorectal cancer (CRC) has increased due to advances in chemotherapy and molecular targeted therapy [1, 2]. However, the clinical benefits of these therapies are often short-lived or restricted to a subpopulation of patients, largely due to the development of distant metastasis and acquired resistance to targeted therapies. In particular, distant metastasis (generally in the liver) is a major reason for cancer-related death [3–5].

We previously cloned KITENIN (KAI1 C-terminal interacting tetraspanin, Vangl1), a gene encoding a membrane-associated, metastasis-enhancing protein. CT-26 mouse colon cancer cells overexpressing KITENIN exhibit increased invasiveness and tumorigenicity and early hepatic metastasis resulting from KITENIN gain of function (KITENIN-GOF) [6], but these effects are suppressed by KITENIN siRNA [7–9]. The functional KITENIN complex promotes cell motility and thereby controls CRC cell invasion, which contributes to metastasis [10]; consistent with this, KITENIN levels are positively correlated with advanced stage [10] and lymph node metastasis [11] in CRC. An unconventional EGFR-independent signal of EGF, the KITENIN/ErbB4–Dvl2–c-Jun axis, also mediates increased CRC cell invasiveness and is associated with acquired resistance to cetuximab [12, 13]. The KITENIN axis also plays an important role in colorectal carcinogenesis within an *APC*-loss environment [14]. Therefore, the KITENIN complex represents a molecular target for therapeutics aimed at blocking the malignant progression of CRC.

To develop novel anti-metastatic agents, we sought to identify therapeutics capable of breaking down the oncogenic KITENIN complex in CRC cells. We reasoned that blocking the KITENIN complex in combination with conventional chemotherapeutics could improve responses in metastatic CRC patients with EGFR/KITENIN overexpression, whose tumors are resistant to cetuximab. Such combination therapy has not been explored previously in CRC; targeting the pathways of distant metastasis and acquired resistance might be essential for complete treatment [15, 16].

In this study, to specifically target the KITENIN complex with new anti-metastatic agents, we searched for low-molecular weight compounds that break down the complex, thereby shutting off its oncogenic signals. We then tested the effectiveness of the identified substances in suppressing colorectal liver metastasis. Here we show that the compound DKC1125 bound the KH-type splicing regulatory protein (KSRP), a downstream factor and stabilizer of the functional KITENIN complex, and specifically suppressed invasiveness of CRC cells expressing higher levels of KITENIN. Thus, DKC1125 specifically blocked oncogenic signals from the functional KITENIN complex in CRC cells and suppressed liver metastasis in CRC with higher KITENIN by targeting the KITENIN complex. Our results suggest that a combination regimen with DKC1125 could be more effective for treating distant metastasis and chemoresistance in CRC patients with high KITENIN expression.

## Methods

### Cell culture and reagents

Cell lines were purchased from the Korean cell line bank (KCLB, Seoul, Republic of Korea) and routinely screened for mycoplasma contamination. CT-26-WT-iRFP-Neo cells were purchased from Imanis Life Sciences (Rochester). Cells were cultured in RPMI-1640 medium or DMEM containing 10% fetal bovine serum (GenDEPOT), 100 units/mL of penicillin, and 100 μg/mL of streptomycin (Corning) at 37 °C in a humidified atmosphere containing 5% CO_2_. Cells were passaged before reaching confluence. Brefeldin A, chloroquine, MG132, 3-MA, rapamycin, and cycloheximide (Sigma) were administered at the indicated concentrations.

### Plasmids and siRNA

Expression constructs for V5-tagged KITENIN, Myc-tagged KSRP deletion or point mutants, His-tagged KSRP bacterial expression deletion mutants, HA-tagged DVL deletion mutants, and GFP-tagged DVL2 deletion mutants were generated by PCR-based methods. All constructs were confirmed by sequencing. pEGFRP-N1-RACK1 was a gift from Anna Huttenlocher (Addgene plasmid #41088). All siRNAs used for gene silencing were purchased from Santa Cruz Biotechnology. Each consisted of a mixture of several sequences, thus eliminating sequence-specific diversity.

### Antibodies and immunoprecipitation

Antibodies against the following proteins were obtained from the indicated suppliers: Dvl1, Dvl3, Lamin A/C, GFP, and tubulin (Santa Cruz Biotechnology); DVL2, Myc, GAPDH, and c-Jun (Cell Signaling Technol); KSRP (Novus); V5 (MBL); HA and Actin (Sigma); KITENIN (Atlas); His and RACK1 (Abcam); and myc-Trap and GFP-Trap (Chromotek). Secondary antibodies were obtained from Thermo Fisher Scientific. For transient transfection analyses, 293 T, Caco2, and HCT116 cells were transfected with various plasmids and harvested for immunoblot analysis 48 h after transfection. For most assays using stable cell lines, mixed polyclonal cells were used to eliminate the effects of clonal variation. Cellular proteins were separated, transferred, and immunoblotted as previously described [6]. Cell lysates from 293 T, Caco2, and HCT116 cells were used for immunoprecipitation experiments as previously described [10].

### Cell viability assay

Viability of Caco2, Caco2/KITENIN-V5, CT-26/EV, CT-26/KITENIN-myc, HCT116, HT29, and 293 T cells was measured by 3-(4, 5-dimethylthiazol-2-yl)-2, 5-diphenyltetrazolium bromide (MTT) assay using the EZ-Cytox Cell viability assay kit (Daeil Lab Service, Korea). Briefly, cells were plated and cultured in 96-well plates (5 × 10^3^ cells/well). After 24, 48, or 72 h, the culture medium was removed, 10 μl of EZ-Cytox reagent was added to each well, and the plates were incubated for another 2 h at 37 °C prior to measurement of cell viability. Absorbance was determined in an ELISA micro-plate reader at a test wavelength of 450 nm.

### Cell invasion assay

Cell invasion was measured using the Transwell migration apparatus as previously described [6]. Briefly, cultured cells were loaded into the top of a 24-well invasion chamber assay plate (Costar). Conditioned DMEM medium containing 10 μg/ml of fibronectin (Calbiochem) and 1% FBS was added to the bottom chamber as a chemoattractant. After 16 or 48 h (HCT116) of incubation, the cells were stained. Cells at the top surface of the filters were wiped off with a cotton ball, and migrated cells on the bottom surface were counted in four random squares of 0.5 mm × 0.5 mm on each filter. The results are represented as the mean ± SEM of the number of cells per field for at least three independent experiments.

### Subcellular fractionation

Cytoplasmic and nucleus fractions were prepared by a subcellular protein fractionation protocol according to the Abcam instructions. Each fraction was resolved by SDS-PAGE and probed for Dvl1/2/3, KSRP, KITENIN, HA, and Myc. Fraction purity was assessed by probing for tubulin or GAPDH for the cytoplasm and Lamin A/C for the nucleus.

### Protein expression and purification

Four KSRP constructs were generated by PCR cloning using full-length KSRP (1–711) in pET-15b as template: KH12 (130–304), KH34 (322–503), KH1234 (130–503), and C-term (492–711). After digestion of the 5′ *Nde*I and 3′ *Xho*I sites with the corresponding enzymes (Promega), the KSRP constructs were cloned into pET-28a. KSRP clones in pET-28a and full-length KSRP in pET-15b were expressed in BL21 (DE3) *E. coli* competent cells and grown at 37 °C in the media containing kanamycin (50 μg/mL) or ampicillin (100 μg/mL), respectively. Protein expression was induced by the addition of 1 mM IPTG (isopropyl β-D-1-thiogalactopyranoside) when OD_600_ reached 0.6–0.8, and the cells were grown overnight at 25 °C. The cells were harvested and homogenized by sonication with lysis buffer (25 mM Tris pH 7.5, 500 mM NaCl, 5 mM imidazole) and centrifuged (15,000 *g*, 30 min, 4 °C) to isolate supernatant containing soluble target proteins. The supernatant was loaded into columns packed with Ni-NTA resin (Qiagen) and the resin was washed with wash buffer (25 mM Tris pH 7.5, 500 mM NaCl, 20 mM imidazole). The target proteins were eluted with elution buffer (25 mM Tris pH 7.5, 500 mM NaCl, 200 mM imidazole) and the eluate was treated overnight at 4 °C with thrombin (HTI) to digest the His-tag. The target proteins were further purified by size exclusion chromatography (SEC; HiLoad 16/60 Superdex 200 pg; GE Healthcare) in column buffer (50 mM Tris pH 7.5, 150 mM NaCl). SEC fractions corresponding to the target molecular weight were collected and dialyzed overnight against column buffer. The proteins were concentrated and used for ITC, GFP/Myc-Trap, and immunoprecipitation.

### Pulldown assay

Ni-NTA bead-bound 6 × His-KSRP was incubated with HCT116 cell lysates and DKC1125 (10 mM) in pulldown buffer [50 mM Tris-Cl (pH 7.5), 1 mM dithiothreitol (DTT), 4% (v/v) glycerol, 0.1 mg/ml BSA, 5 mM MgCl_2_, 1 mM ATP, and 50 mM NaCl] at 4 °C for 2 h. Following incubation, the beads were washed thoroughly using wash buffer (pulldown buffer with 300 mM NaCl) and bead-bound proteins were resolved on SDS-PAGE followed by immunoblot analysis with antibodies against His.

Purified KSRP-His proteins were mixed lysates of HCT116 or Caco2 cells transfected with DDIX-DVL-GFP or RACK-GFP, respectively, in the pulldown buffer described above. Then, GFP-Trap bead and DKC1125 (10 mM) were added (25% slurry) and incubated on a rotator at 4 °C for overnight. Following incubation, the beads were washed three times, and bead-bound proteins were resolved by SDS-PAGE followed by immunoblot analysis with antibodies against KSRP.

### Preparation of DKC1125-immobilized Affigel-10

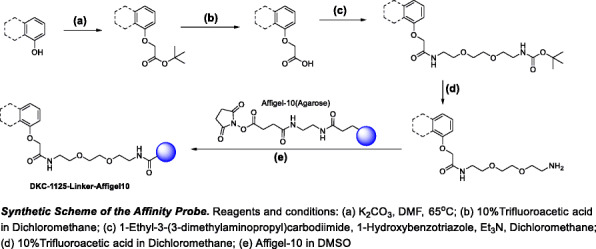



#### Synthesis of DKC1125-TG linker

t-Butyl bromoacetate and potassium carbonate were added to a round-bottom flask with DKC1125 dissolved in DMF. The mixture was stirred at 65 °C for 5 h, and then the reaction mixture was filtered, concentrated, and purified by chromatography to give acetic acid linker-attached DKC1125. A solution of acetic acid linker-attached DKC1125 was treated with 10% trifluoroacetic acid at 0 °C. The reaction mixture was stirred at room temperature (RT) for 30 min and solvent was evaporated. TEA was added to a solution of the acid in dichloromethane, followed by EDCI and HOBt. The mixture was stirred at RT for 15 min, after which the mixture was treated with the TG-Boc amine. The reaction mixture was stirred at RT for 18 h, diluted with EtOAc, and washed with saturated NaCl, dried (Na_2_SO_4_), and concentrated. The resultant crude was adsorbed onto a plug of silica gel and purified by chromatography to provide the product. A solution of t-butyl TG linker-attached DKC1125 was treated with 10% trifluoroacetic acid at 0 °C. The reaction mixture was stirred at RT for 30 min, and the solvent was evaporated to give the amine version of the DKC1125-TG linker.

#### Synthesis of DKC1125-immobilized Affigel-10

Affigel-10 was transferred into a 3 ml cartridge with a polyethylene frit. The supernatant solvent was drained and the Affigel was washed with DMSO. A solution of the free-amine linker version of the DKC1125 in DMSO and DIEA were added to the gel. The cartridge was shaken well for 3 h at RT. The resulting slurry was drained, and the gel was washed with DMSO. The loading level (90%) was determined by analyzing the eluent mixed with an internal standard by LCMS and comparing the result to the initial reaction mixture. A solution of ethanolamine in DMSO and DIEA was added to the reaction cartridge and shaken well for 3 h at RT. The resulting slurry was drained, and the gel was washed with DMSO, water, and 2% sodium azide in water. The Affigel product was stored in 2% sodium azide solution in water at 4 °C.

### Luciferase reporter assay

Two hundred ninety-three T cells (1 × 10^4^) were seeded in 24-well plates and transfected with TOP-flash with pRenillaTK and the indicated plasmid (total DNA, 200 ng) using FuGENE 6. After 48 h, cells were treated as indicated. After 16 h, dual luciferase reporter assays (Promega) were performed on an automated GloMax luminometer (Promega). Reporter activity levels were calculated by normalizing luciferase values against *Renilla* values. All experiments were performed in triplicate and repeated at least twice.

### Autophagosome staining

To determine whether DKC1125 induces autophagy in KITENIN-expressing Caco2 cells, cells were grown on coverslips for 24 h. Cells were washed with PBS and then stained with Cyto-ID green fluorescence reagents (Enzo Life Sciences, Plymouth Meeting, PA, USA) for 1 h at 37 °C in a cell culture incubator. Cells were washed with PBS and mounted with Vectashield mounting medium containing DAPI (Vector Labs). Cells were imaged by confocal microscopy.

### RNA immunoprecipitation and Q-PCR

RNA immunoprecipitation was performed following the Abcam protocol. Briefly, cells were cross-linked with formaldehyde (0.75%) and harvested after washing with 1 × PBS. Nuclei were pelleted by centrifugation and resuspended in RIP buffer [150 mM KCl, 25 mM Tris, pH 7.4, 5 mM EDTA, 0.5 mM DTT, 0.5% NP40, 100 U/mL RNase inhibitor, and proteinase/phosphatase inhibitor cocktail (GenDEPOT)]. Chromatin was sheared by sonication and pelleted by centrifugation. For each IP, 5 μg of KSRP antibody was added, and the samples were incubated overnight at 4 °C with rotation. Protein A/G–agarose beads (Thermo) were added to the samples, which were rotated at 4 °C for 2 h, and then washed three times for 2 min each with RIP buffer. The RNA pellet precipitated with the beads was extracted directly with Trizol-LS (Invitrogen) and resuspended in 20 μl of RNase-free water. Total RNA, including KSRP-binding microRNAs, were reverse-transcribed using a microRNA reverse transcription kit (Qiagen). Mature miR-124 expression was quantified by quantitative PCR (qPCR) using a microRNA primer assay kit (Qiagen). U6 transcript was used as an internal control to normalize RNA input.

### miScript miRNA PCR array

cDNA synthesized using the miRNA RT kit (Qiagen) was applied to the Rotor-Disc 100Format R miScript miRNA PCR array (Qiagen), which contains primers for the detection of 85 cancer pathway or tumor suppressor miRNAs and duplicates of six internal controls. For qRT-PCR, the sample Disc was run on a Rotor-Gene Q (Qiagen), and a global CT mean was calculated for the miRNA targets that were commonly expressed in all samples being analyzed. Among the six control genes used for miRNA expression profiling in individual samples, *SNORD62* was used as an internal control for normalization of qRT-PCR data. Fold change was calculated using the 2^−∆∆CT^ method. Relative fold change values > 1.25 were considered to be meaningful positive changes, whereas values < 0.75 were considered to be meaningful negative changes.

### Isothermal titration calorimetry (ITC)

To measure the binding affinity of the DKC1125 and KSRP constructs, ITC measurements were carried out by affinity-ITC (TA Instruments) or VP-ITC (MicroCal) at 25 °C. DKC1125 was completely solubilized with DMSO (100 mg/mL; 336 mM) and diluted with column buffer to the desired concentration. The sample cell contained KSRP constructs, and the syringe contained DKC1125 with a molar ratio varying from 1:10 to 1:100 (KSRP:DKC1125). The dissociation constant (K_d_) was calculated using the NanoAnalyze (TA Instruments) or Origin software (MicroCal).

### Computational docking model

Because the binding of a compound to a target protein initiates a biological action, it is important to determine the binding pose at the atomic level. In this case, the driving force of ligand–protein binding was a hydrogen bond. According to a possible binding pose of DKC1125, the compound was located between the KH3- and KH4-domains. To construct the KH34-domain of KSRP, the third and fourth KH-domains were obtained from the Protein Data Bank (PDB) using IDs 2HH3 (KH3: residues 318–418) and 2HH2 (KH4: residues 423–525), respectively [17]. Because the conformation of the missing loop and the configuration of KH34-domain are unknown, several potential protein structures with various loop fluctuations were required. Based on the configuration of the KH23-domain (PDB entry 2JVZ), the composition of the KH3- and KH4-domain was aligned using the Swiss-model homology server [18] and then the missing loop (residues 395–424) between the KH3- and KH4-domains was randomly modeled in intensive mode on the Phyre2 server [19]. Using the modeling techniques, possible structures of KH34-domain were produced with various side chain configurations of the missing loop. In comparison with several protein models, the root mean square deviation (*rmsd*) of the backbone was < 3 Å, indicating that all proteins were similar. In addition, because the drug binding site of the KH34-domain is unknown, it was necessary to predict the binding site on the protein surface. Several possible protein structures were run using the GHECOM web server (http://strcomp.protein.osaka-u.ac.jp/ghecom/). Potential binding sites were predicted as site 1 (residues 395–418) in the KH3-domain and site 2 (residues 464–472) in the KH4-domain. According to the results of the binding site prediction, site 2 had a slightly higher probability of being the ligand-binding site. To confirm the binding site of the KH34-domain and select a possible target protein structure, docking simulations were performed using AutoDock Vina [20]. Several models of the KH34-domain were used as the target protein, and the three-dimensional (3D) structure of DKC1125, as the ligand, was built using the MarvinSketch software (Fig. 2c). The searching region was assigned to site 1 of KH3-domain and site 2 of KH4-domain. After the docking calculation, we compared the score of the protein–drug interaction; a lower score indicated stronger binding affinity. Finally, two complex structures were ranked according to lowest score value. The binding site of the two complexes was equivalent to site 2 of the KH4-domain, but the binding modes of the ligand–protein interaction were different. To test the structural stability under physiological conditions, we ran a molecular dynamics (MD) simulation in a solvent environment. The complex system was solvated in a cubic box of TIP3P water model. The protein force field and ligand force field were AMBER14SB [21] and the general AMBER force field (GAFF) [22], respectively. The solvated system was optimized by conjugate gradients and set up for MD simulation. A velocity Verlet algorithm was used to integrate Newton’s equations of motion. Particle-Mesh-Ewald (PME) summation for electrostatic interaction was chosen for the periodic boundary condition. A standard cut-off of 10 Å was set up for neighbor list generation and coulomb and Lennard-Jones interactions. The time step was about 2 fs with the bonds including hydrogen atoms to constrain. For *NPT* ensemble, the temperature was 310 K and the pressure was 1 atm. The thermostate was worked by temperature coupling using velocity rescaling with a stochastic term. Exponential relaxation pressure coupling with a time constant was used. The runtime of *NPT* simulation was about 1 ns for pre-equilibrium. For full equilibrium, *NVT* simulation was performed for 100 ns. The time profiles of potential energy and rmsd of the protein backbone were computed for two possible complexes. After discarding the initial 50 ns, the cluster analysis was run to get the most distributed structure, and the neighborhood side chain contacts for the drug–protein interaction were calculated. All MD simulation and structural analysis were performed using the Gromacs software.

### In vivo tumor growth

All animal experiments were performed under the guidelines of the Chonnam National University Medical School Research Institutional Animal Care Committee, and all experimental protocols were approved by the committee (CNU IACUC-H-2018-65, CNU IACUC-H-2019-6).

Syngeneic mouse tumor models are useful for testing the anticancer effects of candidate substances in short-term studies [7]. Accordingly, we used the CT-26 cell/syngeneic mouse model to investigate the in vivo effects of DKC1125 on colorectal tumorigenesis. Male Balb/c mice (5 weeks old) were purchased from DaMul Science (Korea) and acclimated for 1 week prior to subcutaneous injection of syngeneic CT-26/Vector and CT-26/KITENIN cells (1 × 10^5^) into the dorsum. Tumor volume (*V*) was calculated using the following equation: *V* = 1/2×*a*×*b*^2^, where *a* and *b* are the longest and shortest diameters of the tumor (in millimeters), respectively. Tumor volume was measured every other day for 19 days to verify the effects of DKC1125. All mice were sacrificed after Day 19, and subcutaneous tumor grafts were surgically excised and weighed.

### In vivo hepatic metastases model

Five- to six-week-old Balb/c mice or Balb/c-nu mice were obtained from OrientBio (Seongnam, Korea) and housed in metal cages with free access to water and food. A syngeneic mouse model of colorectal liver metastasis (CLM) was established by infusion of tumor cells into the portal system via intrasplenic injection [23]. In brief, CT-26/KITENIN-iRFP-expressing cells (1 × 10^5^ cells/mouse) or HCT116 cells (3 × 10^6^ cells/mouse) were injected into the spleen of the syngeneic or nude mice, respectively, to induce CLM. Splenectomy was performed 10 min after injection of CT-26 cells. The skin and peritoneum were sutured, and the mice were used to study suppression of tumor growth. Mice were randomly assigned to four groups: vehicle (0.1% DMSO), 5-FU (100 mg/kg in syngeneic mice; 50 mg/kg in nude mice) alone, DKC1125 (5 or 10 mg/kg in syngeneic mice; 5 mg/kg in nude mice) alone, and a combination of 5-FU and DKC1125. For syngeneic mice, 5-FU and DKC1125 were administered via intraperitoneal injection five times over 2 weeks starting 14 days after inoculation of tumor cells. For nude mice, 5-FU and DKC1125 was administered via intraperitoneal injection five times over 2 weeks starting 4 or 6 weeks after inoculation of tumor cells. Metastatic tumor nodules in the liver with a diameter of > 1.0 mm were counted using a microscope, and a metastasis score was assigned based on nodule size, as follows: 0 (no gross metastasis), 1 (tumor size > 1 mm), 2 (tumor size 1 > 5 mm), and 3 (tumor size > 10 mm). The metastasis score was multiplied by the number and score of the nodules. Fluorescence images of liver nodules expressing iRFP were acquired using a fluorescence-labeled organism bioimaging instrument (FOBI; Cellgentek, Korea), and total fluorescence emitted from liver nodules was compared between groups.

### Statistics

Experimental differences were tested for statistical significance by ANOVA followed by Tukey’s HSD post hoc test or Student’s *t* test. All statistical tests were two-sided, and *P*-values of less than 0.05 were considered statistically significant. Statistical analyses were performed using the PASW Statistics 20 software (SPSS, an IBM Company, Chicago, IL).

## Results

### Compound DKC1125 blocks AP-1 activity and cell invasiveness in KITENIN-overexpressing cells

**Fig. 1:**
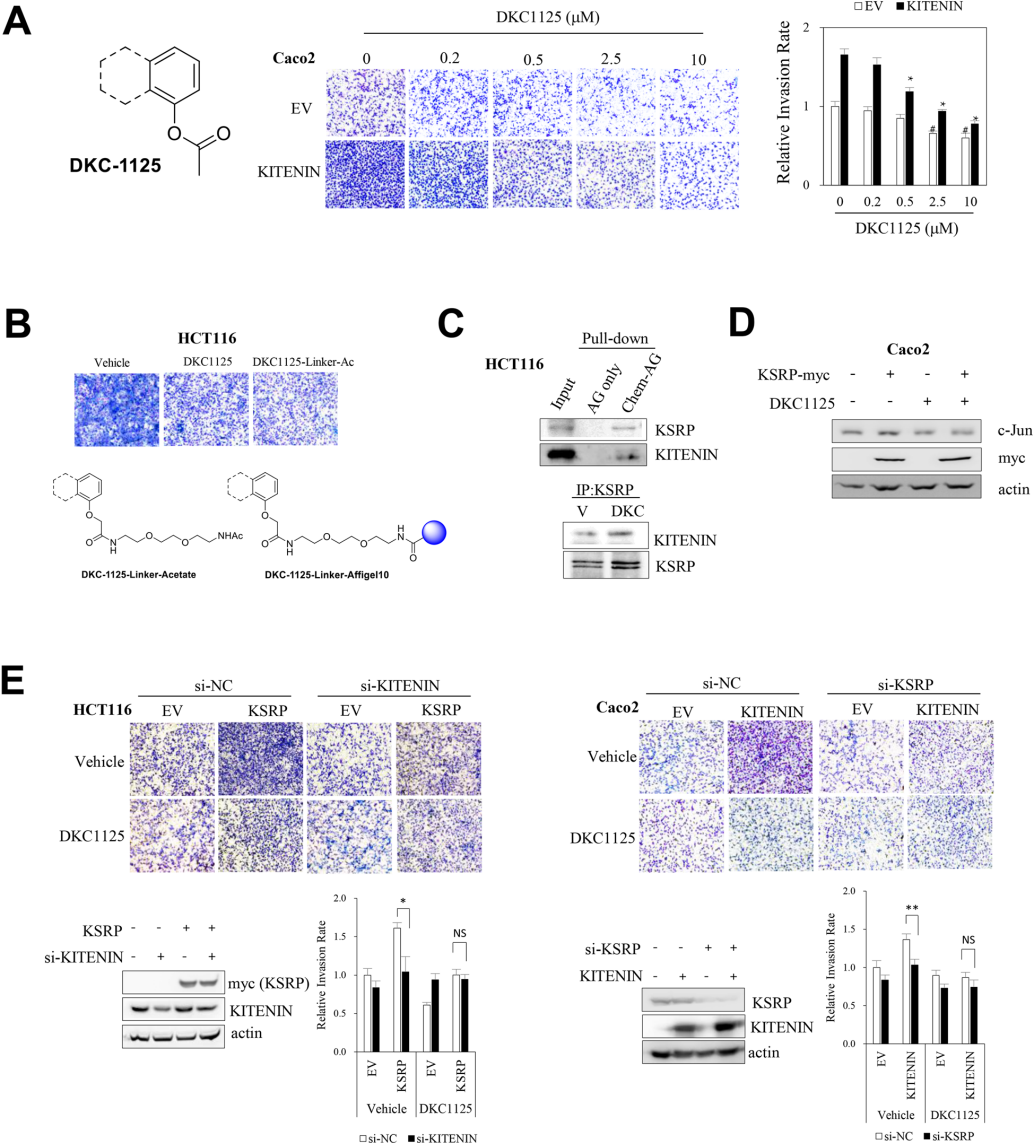
Identification of DKC1125, which suppresses KITENIN gain of function in vitro, by screening a small-molecule library and detection of KSRP, which binds specifically to DKC1125. **a** Structure of DKC1125 and its inhibitory effect on cell invasiveness. After suspending 7 × 10^4^ empty vector-transfected Caco2 CRC cells (Caco2/EV) or KITENIN-transfected cells (Caco2/KITENIN-V5) in medium containing 0.2% BSA, a Transwell invasion assay was performed. Cells were exposed to the compound for 48 h at the indicated concentrations (up to 10 μM). Images represent three independent experiments. The histogram represents invading cells, which were counted in five chosen areas, calculated as rate relative to vehicle-treated control, and represented as bar graphs (mean ± SEM, *n* = 3). The asterisk indicates a significant difference between Caco2/KITENIN-V5 cells (vehicle-treated vs DKC1125-treated; *P < 0.05; **P < 0.01) and Caco2/EV cells (vehicle-treated vs DKC1125-treated; #*P* < 0.05). **b** Structure of chemical probe containing DKC1125. The chemical probe was constructed by covalently attaching Affigel beads to the DKC1125 linker. Cell invasion assay was performed in HCT116 CRC cells, comparing DKC1125-TN-linker-acetate and DKC1125 itself. DKC1125-TN-linker-acetate (DKC1125-Linker-Ac) was constructed by attaching an acetate group to the linker instead of Affigel at the same position as DKC1125, and was used as a positive control for Affigel-bound DKC1125. **c** Detection of KSRP protein through analysis of pull-down proteins with the chemical probe. The chemical probe, prepared as described above, was mixed with the HCT116 cell lysate for pulldown of the chemical-binding proteins, which were verified by immunoblot analysis using antibody against KSRP and KITENIN (upper panel). Immunoprecipitation (IP) was performed with KSRP-specific antibody, and elevated KITENIN binding was observed in the presence of DKC1125 (0.5 μM) (lower panel). **d** Assessment of the downstream effectors of the KITENIN axis after treatment of Caco2 cells with DKC1125 (0.5 μM). **e** Effects of modulation of KSRP on the invasiveness of Caco2 and HCT116 cells, and influence of DKC1125 treatment. Forty-eight hours after transfection of each construct, the increase in invasiveness due to KSRP expression was examined by Transwell assay in si-scrambled (si-NC) or si-KITENIN-expressing HCT116 cells (left panel) or elevated invasiveness by KITENIN expression in si-scr or si-KSRP-expressing Caco2 cells (right panel) with or without DKC1125 (0.5 μM). Data are expressed as in Fig. 1**a**. *p < 0.05, compared with the control group

We previously reported that overexpressing KITENIN promotes activation of AP-1 through interaction with Dishevelled (Dvl)/PKCδ, which drives malignant progression of colon tumors and enhances metastasis [6, 7, 10, 12, 14]. Here, we searched for functional blockers of AP-1 activity in KITENIN-overexpressing cells using a small-molecule compound library containing about 6800 species (provided by Korea Chemical Bank). By screening for suppression of AP-1 activity and AP-1 target gene expression, KITENIN–ErbB4 interaction, and cell invasion of Caco2 CRC and MDA-MB231 breast cancer cells, we identified five candidate compounds (Additional file 1: Fig. S1A). Among them, we chose DKC1125 (Disintegrator of KITENIN Complex #1125) as the most promising. DKC1125 (MW = 297) inhibited AP-1 activity in 293 T cells with an IC_50_ value of 0.48 μM (Additional file 1: Fig. S1B) and suppressed the KITENIN-mediated increase in cell invasion in both Caco2 and MDA-MB231 cells (Additional file 1: Fig. S1C) without affecting cell viability (Additional file 1: Fig. S1D). The structure of DKC1125 is shown in Fig. 1a.

DKC1125 significantly inhibited the increase in cellular invasion caused by ectopic KITENIN expression in Caco2 cells, starting at a concentration of 0.5 μM (Fig. 1a, *p* < 0.05). The effect was stronger in KITENIN-overexpressing cells than in Caco2 cells expressing vector only, which were significantly inhibited by DKC1125 from a concentration of 2.5 μM (*p* < 0.05).

### DKC1125 binds to KSRP, a downstream component of the KITENIN complex

To find a protein whose function is modulated after binding with DKC1125 that also mediates the oncogenic activity of the functional KITENIN complex, we attached Affigel beads to linker-bound DKC1125 (DKC1125-linker-Ac) to prepare a chemical probe. Invasion by HCT116 cells, the CRC cells with the highest endogenous KITENIN levels [12], was inhibited by treatment with 0.5 μM DKC1125-linker-Ac (0.5 μM, Fig. 1b) to a similar extent as with free DKC115, i.e., DKC1125 and linker-attached DKC1125 had the same activity. Hence, we used Affigel bead-bound DKC1125 for the next experiment. By clipping out the probe-binding proteins that showed a different pattern than the control group (Affigel alone) and subsequent protein sequencing (Additional file 1: Fig. S1E), we identified KSRP, a KH-type splicing regulatory protein that bound DKC1125 (Fig. 1c, upper). To verify the protein sequencing data, blot proteins pulled down from HCT116 cell lysates using a chemical probe were detected with a specific antibody. Both KSRP and KITENIN bound to DKC1125. Co-IP confirmed that KSRP was coupled with KITENIN, and this interaction was slightly stronger after treatment with DKC1125 (Fig. 1c, lower).

The single-stranded RNA-binding protein KSRP negatively regulates gene expression via two main post-transcriptional mechanisms: promoting decay of unstable mRNAs and favoring maturation from precursors of distinct sets of microRNAs (miRNAs, miRs), and controlling pleiotropic cellular functions such as the immune response in distinct cell lines [24, 25]. To investigate how DKC1125 affects the function of KITENIN after binding KSRP, we examined the effect of DKC1125 on the level of c-Jun, a downstream effector of the KITENIN axis that is involved in cell invasion [10]. Treatment with DKC1125 decreased the amount of c-Jun. Whereas KSRP overexpression increased the c-Jun level, this effect was negated by DKC1125 (Fig. 1d). These results suggested that DKC1125 may act as a functional inhibitor of the KITENIN axis in regard to cell invasiveness.

Next, we examined the effect of DKC1125 on cell invasiveness induced by overexpression of KSRP or KITENIN. Ectopic expression of KSRP increased the invasiveness of HCT116 cells, and administration of DKC1125 suppressed this elevation of cell invasion. Interestingly, however, KSRP overexpression-mediated cell invasion returned to the basal level when KITENIN was knocked down, and DKC1125 no longer decreased cell invasion under KITENIN knockdown (Fig. 1e, left). The increase in cell invasion by KITENIN overexpression was also reduced by knockdown of KSRP in Caco2 CRC cells. Likewise, DKC1125 no longer decreased cell invasion under KSRP knockdown (Fig. 1e, right). Because KSRP and KITENIN are binding partners of DKC1125, and knockdown of KSRP or KITENIN neutralizes the inhibitory effect of DKC1125 on the increase in cell invasion mediated by KITENIN or KSRP, respectively, we conclude that KITENIN and KSRP participate in a common cell invasion axis that is affected by DKC1125. In mechanistic terms, KITENIN is localized in the membrane, whereas KSRP is dispersed in the cytoplasm and nucleus as an RNA-binding protein; both proteins form a functional complex that modulates cell invasion, which is inhibited by DKC1125.

### DKC1125 mainly docks to the fourth KH-domain of KSRP

**Fig. 2:**
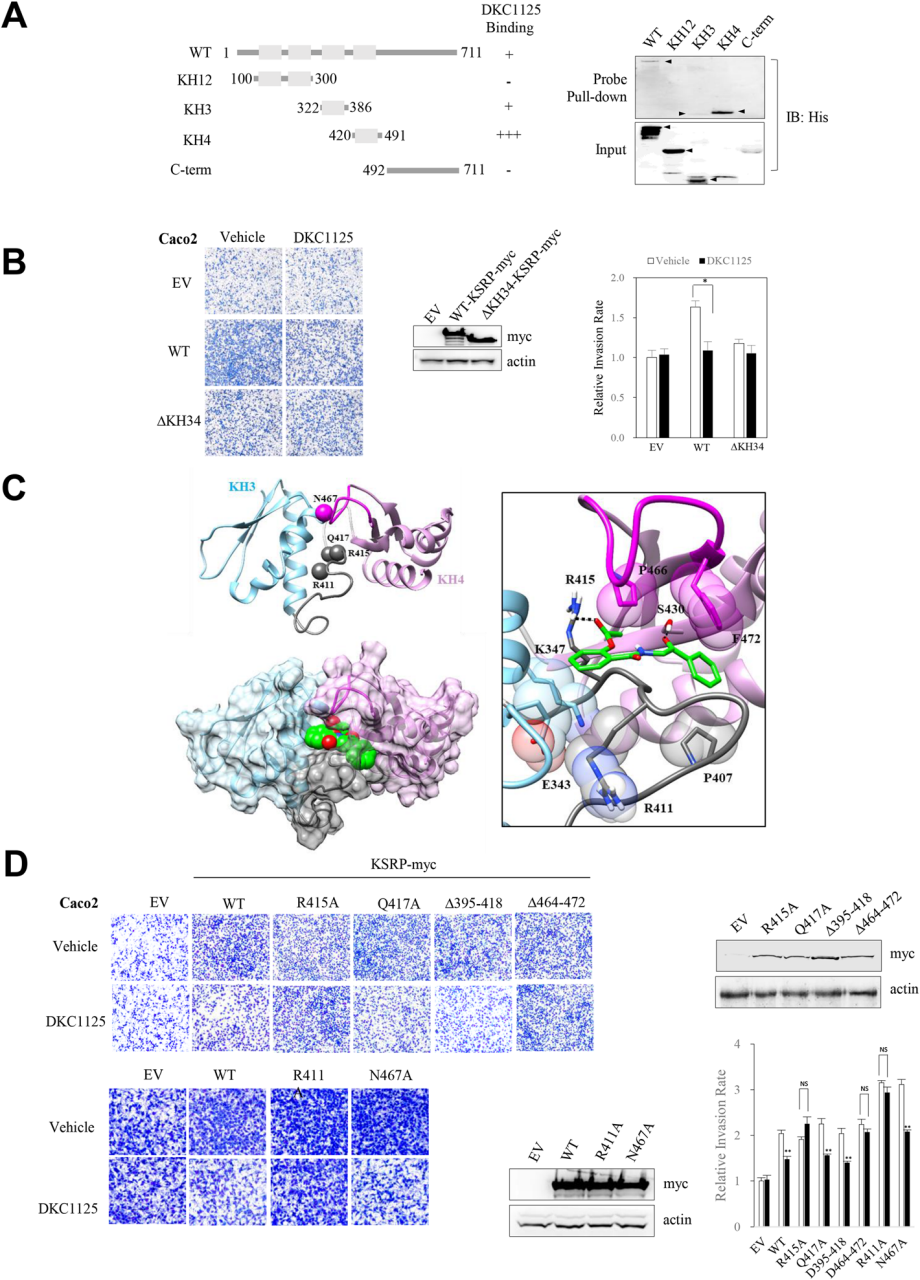
Proposed model of the interaction between DKC1125 and KSRP. **a** DKC1125 interacts with KSRP via the third and fourth KH-domain. Schematic representation of KSRP-His deletion mutants are shown. The four gray boxes indicate the KH-domain of KSRP. Bacterially expressed proteins from His-tagged KSRP deletion constructs were purified with Ni-NTA resin and pulled down with a DKC1125 chemical probe. The interaction was examined by probing the blots with anti-His antibody. Positive bands were detected in KSRP full-length and the KH3 (residues 322–386) and KH4 (residues 420–491) domains, but not in the KH12 domain (residues 100–300) or the C-terminus of KSRP (residues 501–711). Arrows point to the right size of each protein. **b** Effects of wild-type KSRP and KSRP deletion mutants on cell motility and influence of treatment with DKC1125. Cell invasion was compared between Caco2 cells expressing wild-type (WT) KSRP-myc and those expressing KSRP deletion mutant lacking the KH34-domain (ΔKH34-KSRP-myc) after treatment with DKC1125 (0.5 μM). NS: not significant, *p < 0.05. **c** Predicted structure of KH34-domain and a possible binding pose of DKC1125. The sky-blue and pink regions indicate the KH3-domain (residue 324–394) and KH4-domain (residue 425–495), respectively. The random loop between the two domains is colored gray. Two predicted binding sites of the KH34-domain are represented in dark gray (site1; residue 395–418) and magenta (site2; residue 464–472). The spheres represent single point mutations of R411A, R415A, Q417A, and N467A. According to the computational simulation, DKC1125 (green) was put on the KH34-domain and totally covered by site2 of KH4-domain, especially hydrophobic P466 and F472. The main binding force was induced by strong hydrogen bonds (dashed line) between the ligand and protein. DKC1125 formed hydrogen bonds with R415 of the loop and S430 of the KH4-domain. The side chain aggregation of E343, K347, R411, and P407 could stabilize the ligand pocket of the KH4-domain. **d** Effects of KSRP deletion mutant on cell motility, and the influence of treatment of DKC1125. Cell invasion was compared between Caco2 cells expressing wild-type KSRP-myc and the KSRP deletion mutant within the KH34-domain after treatment with DKC1125 (0.5 μM). The amino acids within the putative binding pocket regions predicted to be essential to the binding of DKC1125 by the computer docking model were replaced with alanine or deleted, and the effects of overexpression of the mutants on cell invasion, with or without DKC1125 (0.5 μM), were tested and compared with those of wild-type KSRP. Data are expressed as in Fig. 1**a**. The increase in cell invasion by wild-type KSRP was restored by DKC1125, and the same results were observed in the Q417A, Δ395–418 (site1), and N467A mutant, but not in the R415A, Δ464–472 (site2), or R411A mutant

To determine the precise site of interaction between DKC1125 and KSRP, we incubated deletion mutants with a chemical probe and analyzed pulled-down proteins. Positive bands were detected in KSRP full-length, as well as the KH3 (residues 322–386) and KH4 (residues 420–491) domain. By contrast, the KH12 domains (residues 100–300) and C-terminus of KSRP (residues 501–711) failed to interact (Fig. 2a). Next, we performed isothermal titration calorimetry (ITC) to investigate the binding of DKC1125 with the KH-domains of KSRP, which are RNA-binding targets within KSRP [25]. These experiments confirmed that DKC1125 binds to the third and fourth KH-domains of KSRP (Additional file 2: Fig. S2). We then compared a KSRP deletion mutant lacking the KH34-domain (KSRP-ΔKH34) with wild-type KSRP. Relative to wild-type KSRP, cell invasion was slightly increased by overexpression of KSRP-ΔKH34, and DKC1125 did not affect cell motility induced by KSRP-ΔKH34 (Fig. 2b).

To predict the binding pose of the ligand–protein interaction, we conducted a molecular docking simulation and performed MD simulations to confirm the thermal stability of the complex [20]. Using computational methods, we constructed the KH34-domain and two potential binding-pocket regions were assigned as site1 (residue 395–418) of the loop and site2 (residue 464–472) of the KH4-domain (Fig. 2c, left). DKC1125 formed two strong hydrogen bonds with R415 of the loop and S430 of the KH4-domain, whereas site2 of the KH4-domain surrounded DKC1125, and P466 and F472 of the KH4-domain, a hydrophobic side chain, contacted with the planar amino group and the benzene ring of DKC1125 (Fig. 2c, right). In addition, K347 and E343 of the KH3-domain aggregated with R411 of the loop, decreased fluctuation of the loop, and stabilized the pocket of the KH4-domain. Therefore, DKC1125 was tightly stuck in the predicted site2 binding pocket.

Then, because the configuration of an accurate binding site of the KH34-domain was previously unknown, we performed complementary mutation experiments based on the simulation data. Each mutant was expressed in CRC cells, and changes in cell invasiveness after DKC1125 treatment were compared (Fig. 2d). The increase in cell invasion mediated by wild-type KSRP was restored by DKC1125, and we observed the same results in Q417A, Δ395–418 (site1), or N467A mutant. By contrast, DKC1125 did not diminish the increase in invasion by cells expressing the R415A, Δ464–472 (site2), or R411A mutant (Fig. 2d).

Based on the results of the docking model and the cell invasion assay, we concluded that the binding of DKC1125 with KSRP occurs by insertion of the compound into a binding pocket, such as site2 of the KH4-domain (Fig. 2c, right). Hydrogen bonding of R415 and the interaction of R411 are consistent with the experimental result; however, Q417 and N467 of the KH34-domain are not involved in the interaction with DKC1125.

### DKC1125 abolishes the association of KSRP with Dvl2

**Fig. 3:**
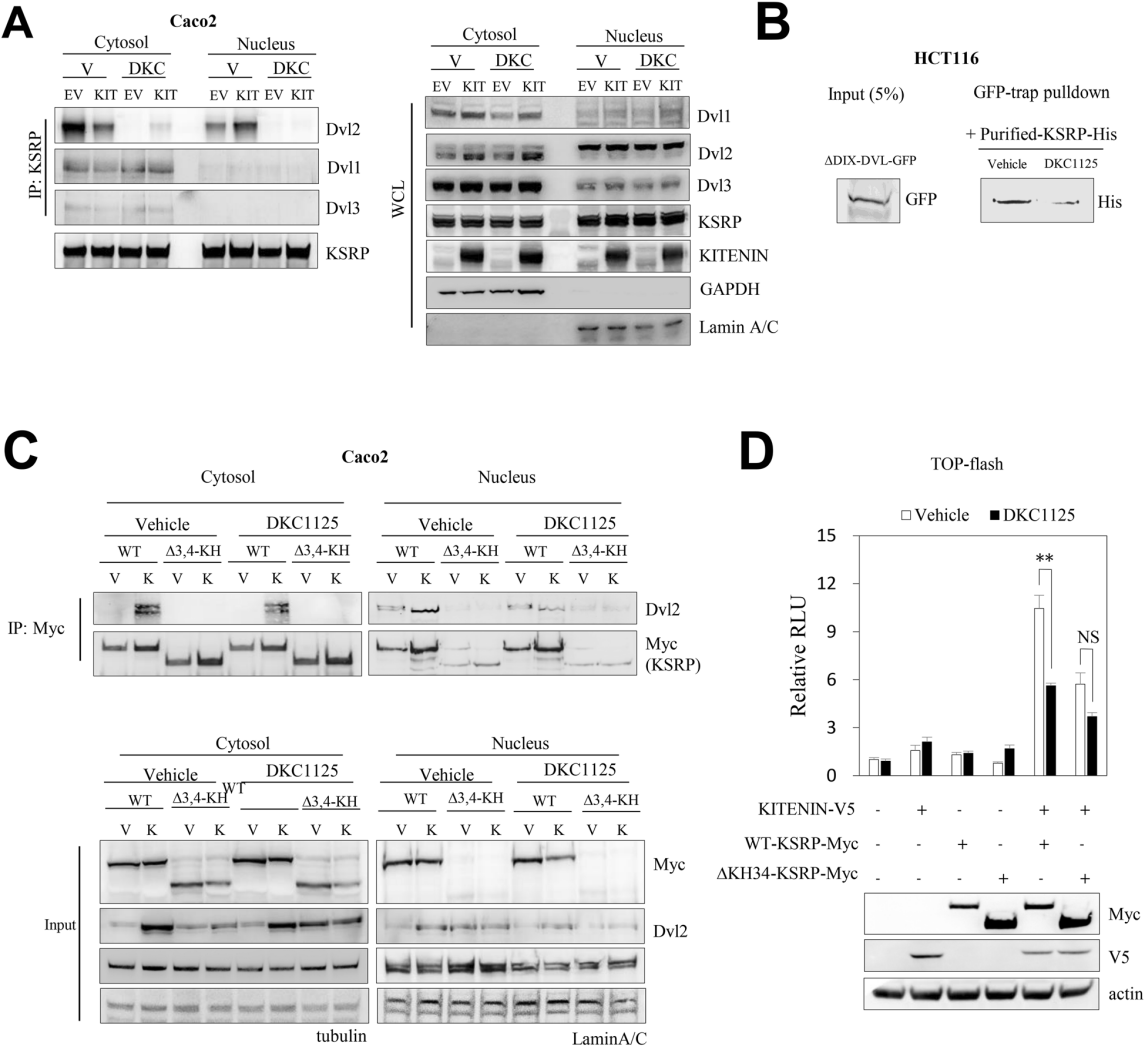
DKC1125 inhibits the function of Dvl by interfering with the interaction of KSRP with Dvl2. **a** Association of KSRP with Dvl2 was inhibited after treatment of DKC1125. Co-IP experiments were performed in Caco2 cells stably expressing EV or KITENIN-V5 using cytoplasmic–nuclear fractionation after treatment with vehicle (V) or DKC1125 treatment (0.5 μM). Precipitates were analyzed by immunoblotting to detect KSRP-Dvl binding. Whole-cell lysate (WCL) of the same pool of cells was co-analyzed as a control. **b** The amount of Dvl2 protein adhering to KSRP was markedly reduced by treatment with DKC1125. Dvl2-ΔDIX-GFP transfected HCT116 cells were collected and lysed, and the supernatants were mixed with purified KSRP-His by Ni-NTA after in vitro bacterial expression and subjected to GFP-Trap. GFP-Trap, consisting of an anti-GFP Nanobody/VHH coupled to agarose beads, was used for effective pulldown of GFP-fusion proteins. Interaction of ΔDIX-DVL2-GFP with bacterially expressed KSRP-His was verified by immunoblotting with anti-His antibody. **c** Effect of DKC1125 on Dvl binding of WT-KSRP or ΔKH34-KSRP. Co-IP analysis was performed using cytoplasmic–nuclear fractions obtained from WT-KSRP or ΔKH34-KSRP transfected Caco2 cells stably expressing KITENIN-V5 or EV. Binding was verified by immunoblotting. **d** Effect of DKC1125 on activation of WNT/β-catenin by expression of KITENIN and/or KSRP. 293 T cells were transfected with the TOP-flash reporter gene and KITENIN-V5, WT-KSRP-myc, or ΔKH34-KSRP-myc, in parallel, and treated with vehicle or DKC1125 (0.5 μM). Luciferase activity was measured at 48 h after transfection and normalized against the activity of TK-Renilla. Data are shown relative to the corresponding TOP-flash value in control cells. Differences in transcriptional activity of TCF/LEF by expression of KITENIN, WT-KSRP, and ΔKH34-KSRP were compared between the presence and absence of DKC1125. Error bars indicate SEM. The asterisk indicates a significant difference between groups (NS, not significant; **P < 0.01)

The effect of KITENIN on cell invasion is mainly due to activation of AP-1 signaling, which is derived from interaction with Dvl/PKCδ [10] as well as from Nrdp1-dependent downregulation of KITENIN-bound phospho-Dvl2 and subsequent upregulation of c-Jun [12, 26]. In addition, KSRP binds Dvl3, and the Dvl3–KSRP interaction regulates canonical Wnt/β-catenin signaling by stabilizing β-catenin mRNA; KSRP acts as a negative regulator of the canonical Wnt/β-catenin signal [27]. Because our prior results suggested that KSRP acts as a component of the functional oncogenic KITENIN axis that modulates CRC cell invasion (Fig. 1e), we investigated whether Dvl affects the oncogenic context of the KITENIN complex through binding KSRP and, if so, whether DKC1125 can modulate these interactions. Like KSRP, Dvl participates in multiple functions by translocating between the cytoplasm and the nucleus [28, 29]. Thus, we investigated KSRP-Dvl binding by fractionating the cytoplasm and nucleus under KITENIN overexpression. DKC1125 blocked the binding of KSRP and Dvl in the cytoplasm and nucleus (Fig. 3a). In particular, among the three isoforms, DKC1125 showed the most potent inhibitory effect on binding of KSRP to Dvl2. Interestingly, although interaction of KSRP with Dvl2 was inhibited by DKC1125, there were no quantitative changes in their levels in the cytoplasm and nucleus in response to DKC1125. In addition, overexpression of KITENIN decreased binding of KSRP-Dvl2 in the cytoplasm but increased interaction in the nucleus. Based on the observation that binding of KSRP to Dvl2 in the nucleus is promoted under KITENIN overexpression, and that DKC1125 abolishes this interaction, we propose that KSRP–Dvl2 interaction mediates KITENIN-GOF through positive regulation of the Wnt/β-catenin signal. Conversely, this KSRP-Dvl2 interaction is important for the KITENIN-induced oncogenic gains of function because DKC1125 inhibits KITENIN-GOF by blocking the binding of KSRP with Dvl2.

Next, we sought to determine which region of Dvl interacts with KSRP in CRC cells. We found that the interaction between KSRP and Dvl2 was mediated by the PDZ domain of Dvl2 (Additional file 3: Fig. S3A). Hence, we sought to confirm the direct binding of KSRP with Dvl2 and the disappearance of this interaction upon treatment with DKC1125. Dvl2-ΔDIX-GFP and KSRP-His bound tightly, and the amount of Dvl2 adhering to KSRP was markedly decreased by treatment with DKC1125 (Fig. 3b). No quantitative change or shift in cytoplasm-to-nucleus of KSRP was observed after expression of each of the domain-deleted mutants of Dvl2 (Fig. 3a). This implies that Dvl2 is regulated by KSRP rather than vice versa.

Because DKC1125 had a blunted inhibitory effect on cell invasion by KSRP-ΔKH34 relative to wild-type KSRP (Fig. 2b), we asked whether the KH34-domain might be involved in the binding of KSRP with Dvl2. KSRP-ΔKH34 did not bind Dvl2 and was present mainly in the cytoplasm, and only rarely in the nucleus (Fig. 3c). As shown in Fig. 3c, the level of Dvl2 protein was increased by wild-type KSRP under KITENIN overexpression, relative to the level of Dvl2 without the KITENIN background. Moreover, expression of the KSRP-ΔKH34 mutant, which neither increased cell invasion nor was affected by DKC1125, did not induce an increase in the level of Dvl2 protein. These results indicate that the effect of KSRP on cell invasion is closely related to the movement of KSRP between the cytoplasm and the nucleus, as well as to the interaction with Dvl2.

If the stability of Dvl is regulated by KSRP, blocking the interaction of KSRP with Dvl by DKC1125 would negatively affect the activity of the WNT/β-catenin pathway by decreasing the level of Dvl. We used the TOP-flash luciferase reporter assay to determine whether decreased KSRP-Dvl2 binding following DKC1125 treatment would affect the level of TCF/LEF transcription. Simultaneous expression of KITENIN and wild-type KSRP resulted in a 10-fold increase in TOP-flash activity, and this increase was significantly inhibited about 50% by treatment with DKC1125. Likewise, co-expression of KITENIN and KSRP-ΔKH34 increased TOP-flash activity, which was roughly one-half of the wild-type KSRP response but was not significantly inhibited after DKC1125 treatment (Fig. 3d). Therefore, binding of KSRP with Dvl under KITENIN overexpression may modulate CRC cell invasiveness through activation of canonical WNT signaling, and blocking the KITENIN-KSRP-Dvl axis by DKC1125 may be due to abrogation of KSRP-Dvl2 binding.

### DKC1125 induces binding of Dvl2 to RACK1 and a decrease in the Dvl2 level under KITENIN overexpression

**Fig. 4:**
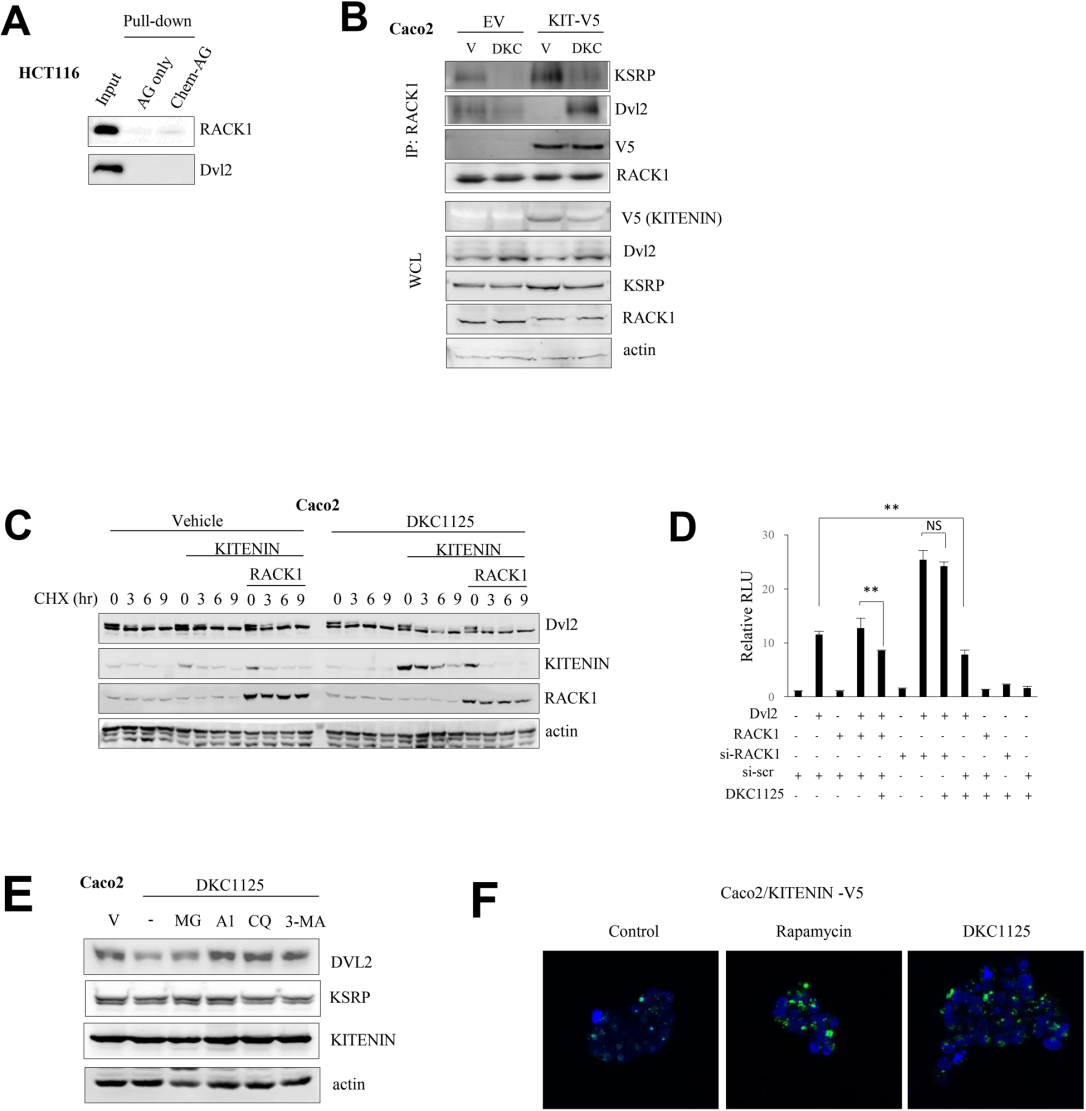
Treatment with DKC1125 results in autophagic degradation of Dvl2 and KITENIN through increased binding to RACK1. **a** RACK1 directly binds to DKC1125. Proteins pulled down by chemical probe using HCT116 lysates were verified by immunoblot analysis using antibodies against RACK1 and Dvl2. **b** Interactions of RACK1 with KSRP or Dvl2 within the functional KITENIN complex following DKC1125. Caco2 cells were transfected with empty vector (EV) or KITENIN-V5 (KIT-V5), and treated with vehicle (V) or DKC1125 (DKC) (0.5 μM). The cell lysates were immunoprecipitated with anti-*RACK1* antibody and immunoblotted with anti-KSRP or anti-Dvl2 antibody. **c** Levels of Dvl2 and KITENIN are reduced more after DKC1125 treatment under RACK1 expression. Caco2 cells were transfected with empty vector (EV) or KITENIN-V5, or co-transfected with KITENIN-V5 and RACK1-GFP, and then treated with vehicle or DKC1125 (0.5 μM). The protein levels of Dvl2 and KITENIN were checked after treatment with cycloheximide at the indicated times. **d** RACK1 affects the activation of canonical WNT signaling by Dvl2. 293 T cells were transfected with the TOP-flash reporter gene and Dvl2, RACK1, or si-RACK1, in parallel, and treated with vehicle or DKC1125 (0.5 μM). Differences in transcriptional activity of TCF/LEF by Dvl2, alone or in combination with RACK1 overexpression or knockdown, were compared between the presence or absence of DKC1125. **e** Autophagic degradation of Dvl2 and KITENIN by DKC1125. Caco2 cells were initially pretreated with vehicle, the proteasome inhibitor MG132 (MG, 10 μM), the lysosomal degradation inhibitors bafilomycin A1 (A1, 100 nM) and chloroquine (CQ, 10 μM), or the autophagosome blocker type III phosphatidylinositol 3-kinase inhibitor (3-MA, 1 mM), and later treated with a high concentration of DKC1125 (5 μM). Levels of Dvl2 and KITENIN were examined by immunoblot analyses. **f** Staining of autophagosomes in DKC1125-treated cells. Autophagosomes were stained in stably KITENIN-expressing Caco2 cells after 24 h treatment with DKC1125 using CYTO-ID autophagy detection dye. Rapamycin was included as a positive control for autophagic induction

Several studies have described the inhibitory effect of KITENIN (Vangl1) on WNT/β-catenin signaling [30], and we also reported the role of E3 ligase Nrdp1 on Dvl degradation under KITENIN overexpression, which is stimulated by EGF [26]. Recent work showed that Vangl2, which is an isotype of KITENIN, binds with receptor for activated C-kinase 1 (RACK1) to inhibit canonical WNT/β-catenin signaling [31]. RACK1 acts as a versatile hub in cancer by shuttling proteins around the cell, anchoring proteins at particular locations, and stabilizing protein activity through interactions with the ribosomal machinery, several cell surface receptors, and proteins in the nucleus [32, 33]. In addition, RACK1 inhibits WNT activity by promoting the autophagic degradation of Dvl [34]. Among the partners of RACK1, KSRP binds with RACK1 to regulate mRNA and miRNA processing [35]. Hence, we investigated whether RACK1 binds to each component of the KITENIN complex and how DKC1125 affects these interactions. Pull-down assay of chemical probe using HCT116 lysate and subsequent anti-RACK1 antibody detection revealed that RACK1 binds DKC1125 but also a member of the functional KITENIN-KSRP complex (Figs. 4a and Fig. 1c). To identify the RACK1- or KITENIN-binding site of KSRP, we performed co-IP in Caco2 cells using different myc-tagged deletion mutants of KSRP and GFP-tagged RACK1 or V5-tagged KITENIN. Only proteins with a KH12-domain retained the ability to bind RACK1. However, KSRP proteins with a C-terminal domain bound with KITENIN (Additional file 3: Fig. S3B). IP experiments showed that the KSRP-RACK1 interaction was stronger under KITENIN overexpression, but this association was lost after treatment with DKC1125. By contrast, under KITENIN overexpression, the interaction between RACK1 and Dvl2 was markedly stronger following treatment with DKC1125 (Fig. 4b).

Considering that the KSRP-Dvl2 and KSRP-RACK1 interactions were attenuated by DKC1125 treatment, whereas RACK1-Dvl2 binding was increased, we speculated that binding of DKC1125 with the KH34-domain of KSRP would release Dvl2 from KSRP but simultaneously stimulate binding of released Dvl2 to RACK1. In particular, these changes in interaction were induced under KITENIN overexpression.

To test the stability of Dvl2 after KSRP-Dvl2 binding was disrupted by DKC1125, we monitored the amount of Dvl2 after treatment with cycloheximide, a reagent that inhibits protein synthesis, in the presence or absence of RACK1. The results revealed that expression of both KITENIN and RACK1 slightly decreased the amount of Dvl2. However, when DKC1125 was administered under these conditions, the level of Dvl2 was further decreased (Fig. 4c). To confirm the role of RACK1-mediated regulation of Dvl2 as a component of the functional KITENIN complex, we performed the TOP-flash reporter assay. With Dvl2 expression alone, we observed a 10-fold increase in TOP-flash activity. By contrast, there was no further increase of TOP-flash activity when Dvl2 was co-expressed with RACK1, but we observed an additive increase of 2-fold or more upon expression of si-RACK1. Treatment with DKC1125 suppressed the activation of TOP-flash by expression of Dvl2 alone or co-expression of Dvl2 and RACK1, but had no effect on the suppression of activated TOP-flash when RACK1 was depleted by treatment with si-RACK1 (Fig. 4d). Thus, these results indicated that downregulation of Dvl2 after treatment with DKC1125 was due to the interaction between Dvl2 and RACK1.

### Elevated levels of RACK1 induce autophagic degradation of Dvl2 and KITENIN

RACK1 can promote the degradation of Dvl2 through autophagy [34]. To test whether degradation of Dvl2 by DKC1125 depends on the induction of autophagy through binding of Dvl2 with RACK1, or on other proteolytic pathways, we performed a parallel assay in which we treated cells with inhibitors of proteasomal or lysosomal degradation [36]. As shown in Fig. 4e, a high concentration of DKC1125 (5 μM) strongly induced degradation of Dvl2, but this was reversed by the lysosomal inhibitors bafilomycin A1 (100 nM) and chloroquine (10 μM) and the autophagosome blocker 3-MA (1 mM), but not by the proteasome inhibitor MG132 (10 μM). This observation suggests that DKC1125 uses RACK1 to degrade Dvl2 via autophagy (Fig. 4e).

Next, to confirm the induction of autophagy by DKC1125, we stained autophagosomes in stably KITENIN-expressing Caco2 cells using CYTO-ID dye. Treatment with DKC1125 for 24 h markedly increased CYTO-ID staining relative to the vehicle-treated control. The number of CYTO-ID puncta per cell and individual puncta size were elevated in response to DKC1125 to levels similar to those in cells treated with rapamycin, a potent inducer of autophagy (Fig. 4f). Interestingly, levels of autophagosomes similar to those after treatment with DKC1125 (5 μM) were formed when RACK1 was overexpressed (data not shown). In addition, the RACK1 protein was upregulated by DKC1125 treatment in several CRC cell lines, whereas the level of Dvl2 decreased (Additional file 3: Fig. S3D). Moreover, treatment with DKC1125 following expression of RACK1 induced the degradation of KITENIN (Additional file 3: Fig. S3E). This finding suggests that RACK1 might control the level of KITENIN via autophagy-dependent degradation. After co-expression of RACK1 and KITENIN, treatment with DKC1125 accelerated degradation of KITENIN; this degradation was attenuated by bafilomycin A1 or chloroquine, but not by MG132 (Additional file 3: Fig. S3E). Thus, RACK1 seems to serve as an adaptor protein for the molecules involved in the downstream signaling of the KITENIN complex.

### miR-124 participates in degradation of the KITENIN complex by DKC1125

**Fig. 5:**
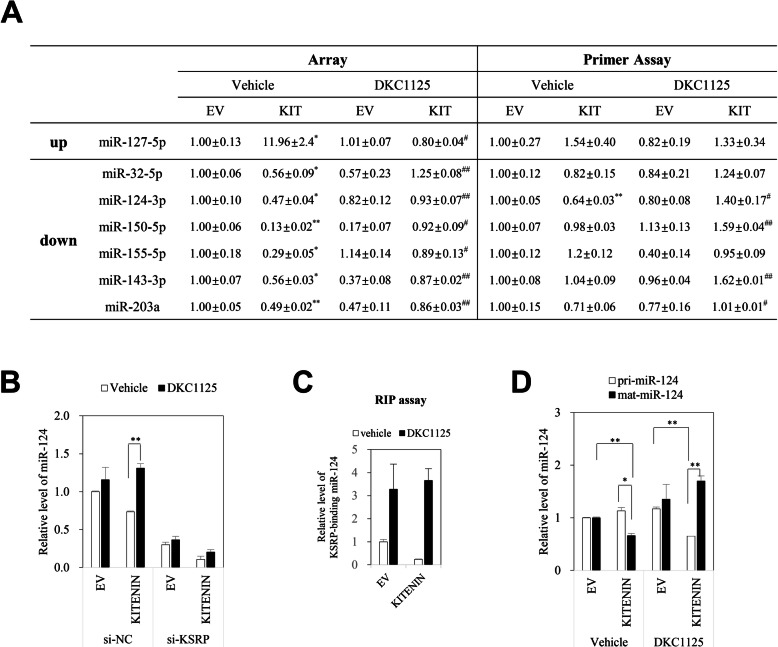
miR-124 also participates in inhibiting the oncogenic KITENIN complex by DKC1125. **a** Modulated miRNA under KITENIN overexpression and effects of DKC1125. Changes in miRNA in Caco2 cells transfected with empty vector (EV) or KITENIN, and the effects of DKC1125 on these cells, were examined by using the Pathway-focused miScript miRNA cancer pathway. After array processing and normalization of raw array data, we identified seven differentially expressed miRNAs whose levels were changed under KITENIN overexpression but restored after treatment with DKC1125 (0.5 μM). miRNAs identified by array were analyzed again by miScript primer assay, which detected the expression of miRNAs under the same conditions as the array. Data were represented as mean ± SEM (*n* = 3). The asterisk indicates a significant difference between Caco2/EV cells and vehicle-treated Caco2/KITENIN-V5 cells (*P < 0.05; **P < 0.01), and a significant difference in Caco2/KITENIN-V5 cells after treatment with DKC1125 (^#^*P* < 0.05; ^##^*P* < 0.01). **b** Effect of DKC1125 on miR-124-3p in KITENIN-overexpressing cells was dependent on the level of KSRP. Caco2 cells stably transfected with empty vector (EV) or KITENIN-V5 were transfected with si-NC or si-KSRP, and then treated with vehicle or DKC1125 (0.5 μM). miR-124-3p transcript levels were compared among groups (right panel). **c** Treatment with DKC1125 increases KSRP-bound miR-124-3p. The anti-KSRP antibody-bound miR-124-3p was examined by RNA immunoprecipitation (RIP) assay in empty vector (EV) or KITENIN-V5-transfected Caco2 cells, which were treated with vehicle or DKC1125 (0.5 μM). **d** Treatment of DKC1125 increases mature miR-124-3p. Caco2 cells stably transfected with empty vector (EV) or KITENIN-V5 were treated with vehicle or DKC1125 (0.5 μM). The unprocessed pri-miR124-3p and mature miR-124-3p were detected and compared among groups

KSRP acts as a regulatory switch and controls context-specific gene expression by promoting the decay of unstable mRNAs and favoring maturation of selected miRNAs [24, 25]. The RNA-binding feature of KSRP functions not only in the nuclear maturation of pri-miRNAs to pre-miRNAs but also in the cytoplasmic maturation of pre-miRNAs into miRNAs, thus representing a link between nuclear and cytoplasmic events [17, 25]. Using miRNA PCR arrays, we examined changes in miRNA under KITENIN overexpression and how DKC1125 affected them (Additional file 7: Table S1). After array processing and normalization of raw array data, we initially chose seven differentially expressed miRNAs whose levels were considerably changed (> 40%) under KITENIN overexpression but restored after treatment with DKC1125. Six of these miRNAs (miR-32-5p, miR-124-3p, miR-150-5p, miR-155-5p, miR-143-3p, and miR-203a) were significantly downregulated, and the other (miR-127-5p) was significantly upregulated in KITENIN-overexpressed cells, however, these changes were significantly recovered after treatment with DKC1125 (Fig. 5a, left). We used the miScript primer assay to validate these findings and obtained results consistent with the array data for three miRNAs (miR-32-5p, miR-124-3p, and miR-203a) in which only miR-124-3p was significantly decreased (*p* < 0.01, Fig. 5a, right). In addition, miR-143-3p and miR-150-5p, which are suppressor miRNAs in CRC, were significantly upregulated after treatment with DKC1125 (> 60%, Fig. 5a, right). However, miR-32-5p and miR-203a were discarded because their endogenous levels were too low. Because KITENIN is a target of miR-124 and is negatively regulated by miR-124-3p [37], we monitored the level of miR-124-3p after reduction of KSRP or overexpression of KITENIN. Consistent with the array results, miR-124-3p levels were decreased by overexpression of KITENIN and rescued by treatment with DKC1125, but this effect was negated by transfection of si-KSRP (Fig. 5b). These results indicated that KSRP, as a component of the KITENIN complex, is essential for regulation of miR-124-3p expression, and that DKC1125 inhibits KSRP function under the control of the functional KITENIN axis.

Next, we performed an RNA immunoprecipitation assay to examine the maturation of miR-124-3p by KSRP (Fig. 5c). The level of miR-124-3p bound by anti-KSRP antibody was markedly elevated in the vehicle-treated control group following DKC1125 treatment, decreased under KITENIN overexpression, and restored by DKC1125 treatment (Fig. 5c). In addition, in KITENIN-overexpressing cells, mature miR-124-3p was upregulated after treatment with DKC1125, whereas pri-miR124-3p, the unprocessed form, was downregulated (Fig. 5d).

After studying the roles of DKC1125 in the functional KITENIN axis, we next explored the effects of DKC1125 on reversing KITENIN-mediated oncogenesis.

### Suppression of cell invasiveness by DKC1125 is dependent on the levels of RACK1 and miR-124

**Fig. 6:**
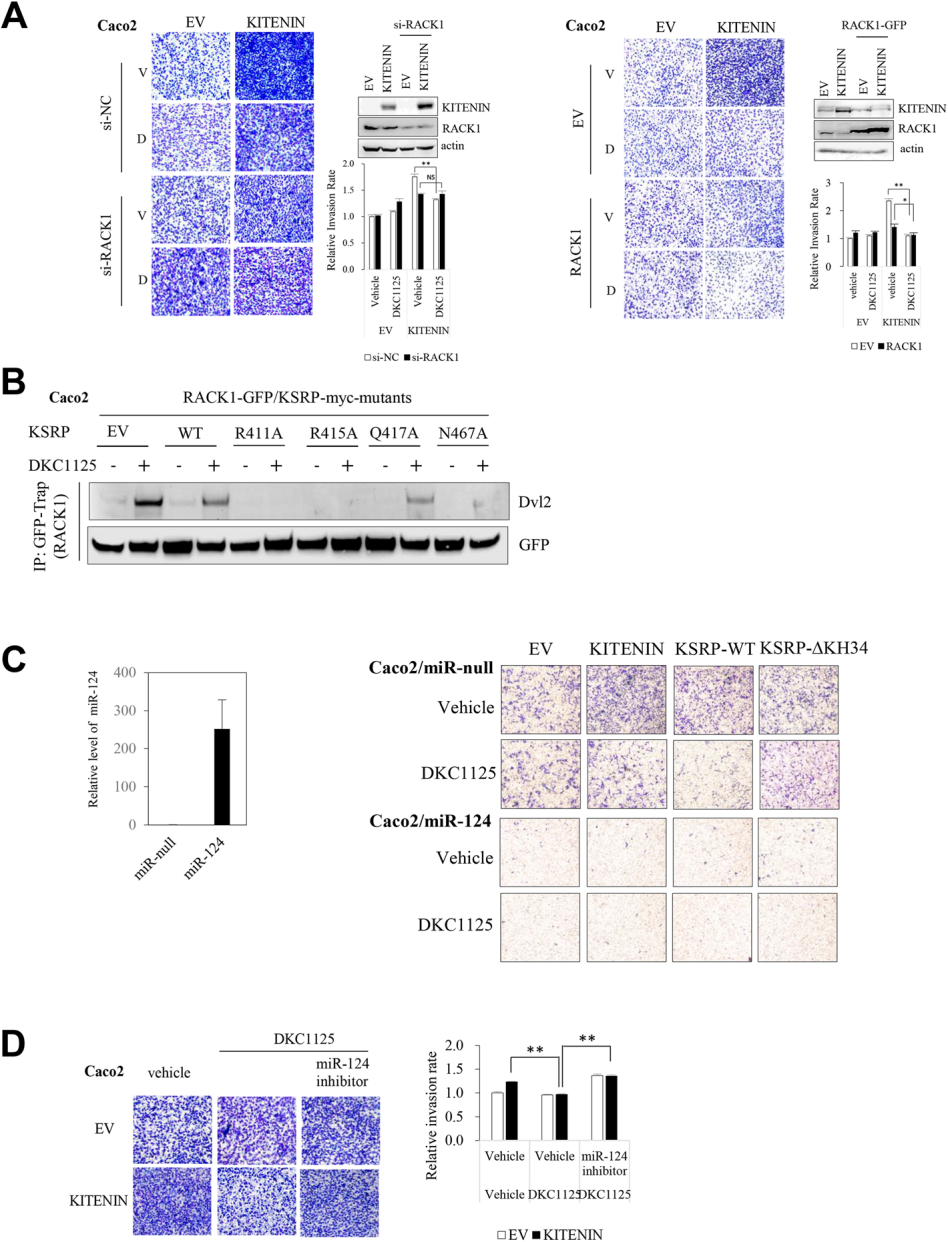
RACK1 and miR-124 are required for the suppressive effects of DKC1125 on cell invasiveness. **a** RACK1 in the KITENIN complex plays a major role in the inhibition of cell invasion by DKC1125. Cell invasion was examined in empty vector (EV)- or KITENIN-transfected Caco2 cells after knockdown of RACK1 via siRNA transfection (left panel), or under ectopic expression of RACK1 (right panel) after treatment with vehicle (V) or DKC1125 (D) (0.5 μM). Data are expressed as in Fig. 1**a**. **b** Dvl2 downregulation by DKC1125 was associated with elevated binding of Dvl2 to RACK1. Several deletion mutants within the binding site of KSRP to DKC1125 were designed and co-expressed in Caco2 cells with RACK1-GFP. Dvl2–RACK1 binding was examined using GFP-Trap and immunoblot analysis after treatment with DKC1125 (0.5 μM), and compared with that of empty vector (EV) or wild-type (WT) KSRP expression. An increase in Dvl2–RACK1 interaction was observed after DKC1125 treatment in cells expressing the Q417A-KSRP or N467A-KSRP mutant, but not in cells expressing the R411A-KSRP or R415A-KSRP mutant. **c** Modulation of miR-124 is also involved in increased cellular invasiveness by the functional KITENIN complex. Detection of transcript of miR-124-3p in stably miR-null- or miR-124-transfected Caco2 cells (left panel). Cell invasion was examined in Caco2 cells stably expressing the miR-null vector or miR-124 that were transfected with the empty vector (EV)-, KITENIN-, WT-KSRP-, or Δ34KH-KSRP, and treated with vehicle or DKC1125 (0.5 μM) (right panel). Data are expressed as in Fig. 1**a**. **d** Inhibitor of miR-124-3p significantly restored the inhibitory effect of DKC1125 on the KITENIN-mediated increase in cell invasion. Cell invasion was compared in empty vector (EV)- or KITENIN-transfected Caco2 cells treated with vehicle or DKC1125, or co-treated with DKC1125 (0.5 μM) and a synthetic-oligo inhibitor of miR-124-3p (50 nM). The asterisk indicates a significant difference in Caco2/KITENIN-V5 cells after treatment with DKC1125, and a significant difference in DKC1125-treated Caco2/KITENIN-V5 cells after treatment with a synthetic-oligo inhibitor of miR-124-3p (**P < 0.01). Data are expressed as in Fig. 1**a**

From the previous results (Fig. 4b), we hypothesized that overexpressed KITENIN promotes the KSRP-RACK1 and KSRP-Dvl2 interactions, and that these components form the functional KITENIN complex. Furthermore, the interactions within the functional complex could result in KITENIN-GOF and thereby organize the specific cellular context in CRC cells that causes malignant tumor progression and promotes distant metastasis. We thus tried to assess the relevance of the interaction of these components within the KITENIN complex and how DKC1125 affects their resultant cellular functions. We first examined cell invasion after altering the expression of RACK1. When endogenous expression of RACK1 was reduced in Caco2 cells via si-RACK1 transfection, DKC1125 treatment had no inhibitory effect on cell invasion (Fig. 6a, left). Ectopic expression of RACK1, however, led to an enhanced effect of DKC1125 (Fig. 6a, right). To determine whether downregulation of Dvl2 by DKC1125 was associated with binding of Dvl2 with RACK1, we examined the Dvl2-RACK1 interaction in Caco2 cells expressing KSRPs with mutated amino acids within the DKC1125 binding site. In cells expressing the Q417A-KSRP or N467A-KSRP mutant, which preserves the binding site of DKC1125, a stronger Dvl2–RACK1 interaction was observed after DKC1125 treatment. However, in cells expressing the R411A-KSRP or R415A-KSRP mutant, which did not interact with the compound, no Dvl2-RACK1 interaction was observed after DKC1125 treatment relative to that in cells expressing empty vector (EV) or wild-type KSRP (Fig. 6b, Additional file 3: Fig. S3C). These results confirmed that treatment with DKC1125 downregulated Dvl2 through increased interaction between Dvl2 and RACK1. Together, these data suggest that although RACK1 itself suppresses CRC cell motility, RACK1 is a critical component of the functional oncogenic KITENIN complex, and that the suppressive effect of DKC1125 on cell invasiveness depends on the expression status of RACK1.

We next examined how the level of miR-124 affects the action of DKC1125 on cell invasiveness by an invasion assay using stably miR-124-3p-expressing cells. miR-124-3p expression in the stable cell line was confirmed by miScript primer assay (Fig. 6c, left). In cells stably expressing the miR-null vector, the increase in invasiveness following KITENIN-V5 or KSRP-myc transfection was diminished by DKC1125 treatment (Fig. 6c, right). However, in miR-124-3p stable cells, no changes in cell invasion occurred regardless of overexpression of KITENIN or KSRP or treatment with DKC1125, indicating that miR-124-3p is an important effector of the increase in cellular invasiveness induced by the functional KITENIN axis. This finding is supported by the observation that a synthetic-oligo inhibitor of miR-124-3p abolished the effect of DKC1125 on the KITENIN-mediated inhibition of cell invasion in Caco2 cells (Fig. 6d). Thus, KSRP and KITENIN maintain cell invasiveness by regulating the expression of miR-124. Taken together, these observations imply that miR-124-3p modulates the KITENIN-KSRP axis, in which its own expression is also regulated, and the increase in mature miR-124-3p by DKC1125 negatively regulates the KITENIN complex and thereby inhibits the invasiveness of CRC cells expressing higher levels of KITENIN (KITENIN-GOF).

Because miR-124-3p plays a suppressor role in the functional KITENIN axis (Fig. 6c), we used a tumor suppressor PCR array to detect suppressor miRNAs that were downregulated in CRC cells expressing higher levels of KITENIN, but that were restored after DKC1125 (Additional file 8: Table S2). In addition to miR-124-3p, the following suppressor miRNAs were more downregulated in CRC cells expressing higher levels of KITENIN than in parental cells (> 25%), as summarized in Table S3 (Additional file 9): miR-100-5p, miR-125a-5p, miR-125b-5p, miR-140-5p, miR-218-5p, miR-34c-5p, miR-98-5p, miR-101-3p, miR-133a-3p, miR-216b-5p, miR-34b-3p, miR-486-5p, miR-502-5p, and miR-622. Based on these results, we conclude that high levels of KITENIN modulate the function of KSRP on the regulation of miRNA biogenesis in CRC cells through binding of KITENIN to the C-terminal domain of KSRP. This affects the interaction of KSRP with other RNA-binding proteins, resulting in specific altered expression of several mature miRNAs. These altered suppressor miRNAs may contribute to organization of a specific cellular context by the KITENIN complex, which has a critical impact on cancer progression, e.g., the elevated invasiveness and metastasis observed in KITENIN-overexpressing CRC cells. In this regard, DKC1125 could reverse KITENIN-mediated oncogenesis by normalizing expression of the deregulated suppressor miRNAs.

### DKC1125 restores the chemosensitivity of 5-FU and oxaliplatin but also the inhibitory action of cetuximab on cell invasion in KITENIN-overexpressing CRC cells

**Fig. 7:**
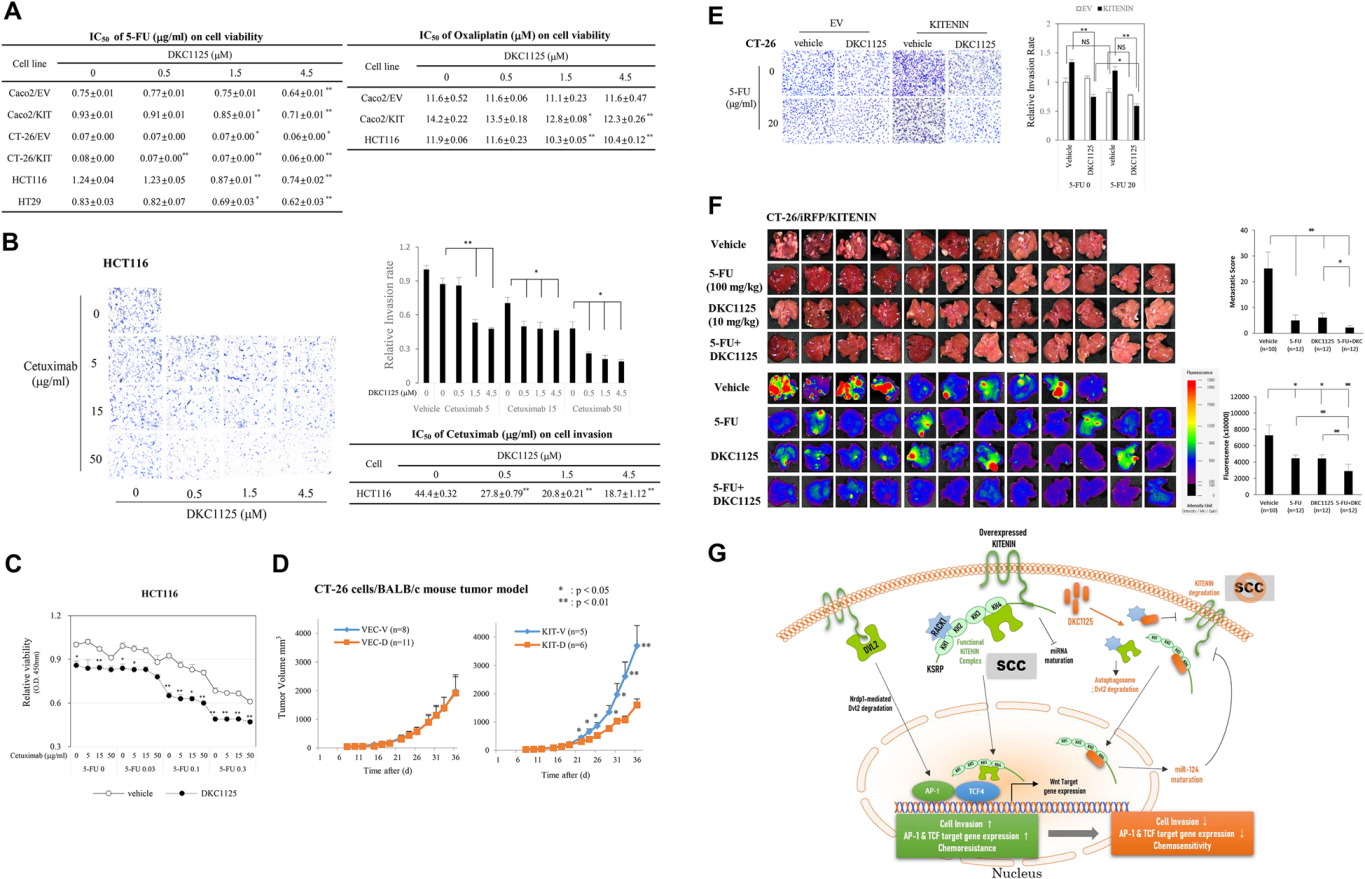
DKC1125 restores sensitivity to 5-FU, oxaliplatin, and cetuximab in KITENIN-overexpressing CRC cells and suppresses hepatic metastasis of CRC. **a** CRC cells overexpressing KITENIN had a blunted chemotherapeutic response to 5-FU and oxaliplatin, and treatment of DKC1125 enhances the inhibitory effects of 5-FU and oxaliplatin on cell survival. Various CRC cells were seeded at 5 × 10^3^ cells/well on 96-well plates, and the cytotoxicity of various concentrations of 5-FU or oxaliplatin and/or DKC1125 in these cells was examined by tetrazolium salt (WST-8) assay. The half-maximal inhibitory concentration (IC_50_) of 5-FU or oxaliplatin alone, or combined treatment of 5-FU or oxaliplatin with DKC1125, was obtained from cell cytotoxicity data at 48 h, as shown in Fig. S5. Data were represented as mean ± SEM (*n* = 3). The asterisk indicates a significant difference of changes after treatment with DKC1125, compared with non-treated IC_50_ value (^*^P < 0.05; ^**^P < 0.01). **b** Treatment of DKC1125 enhances the inhibitory effects of cetuximab on cell invasiveness. Cell invasion was examined in the HCT116 cells treated with vehicle, cetuximab (5, 15, 50 μg/ml), or the combination of DKC1125 (0.5, 1.5, 4.5 μM) and 5-FU (5, 15, 50 μg/ml). Data are expressed as in Fig. 1**a** and Fig. 7**a**. IC_50_ values were represented as mean ± SEM (*n* = 3). The asterisk indicates a significant difference of changes after treatment with DKC1125, compared with non-treated IC_50_ value (^*^P < 0.05; ^**^P < 0.01). **c** Increased cytotoxicity by treatment with DKC1125 in HCT116 cells under combined treatment with 5-FU and cetuximab. HCT116 cells were seeded at 5 × 10^3^ cells/well on 96-well plates, and the effects of DKC1125 on cytotoxicity under combined treatment with various concentrations of 5-FU (0.03, 0.1, or 0.3 μg/ml) and cetuximab (5, 15, or 50 μg/ml) were examined by tetrazolium salt (WST-8) assay 48 h after seeding. Results were shown in line graphs as mean ± SEM (*n* = 3), and compared between groups treated or not treated with DKC1125 (1 μM). The asterisk indicates a significant difference of changes after treatment with DKC1125 in each combined dosage of 5-FU and cetuximab (^*^P < 0.05; ^**^P < 0.01). **d** Effect of treatment with DKC1125 on tumor formation of CT-26 cells in a syngeneic mouse xenograft model. BALB/c mice were injected subcutaneously with 1 × 10^5^ CT-26/EV or CT-26/KITENIN-V5 cells, and randomly sorted into two groups (n = number of mouse) treated with vehicle (0.1% DMSO, indicated as V) or 5 mg/kg DKC1125 (indicated as D) starting 7 days after cell injection. Tumor volumes are represented as means ± SEM. An asterisk in a line graph indicates a significant difference between groups (vehicle-treated vs DKC1125-treated; *p < 0.05, **p < 0.01). **e** Treatment with DKC1125 increases the inhibitory effects of 5-FU on cell invasion by KITENIN-overexpressing CT-26 cells. Cell invasion was compared between CT-26 cells transfected with empty vector (EV) or KITENIN and treated with vehicle, DKC1125 (0.5 μM), or the combination of DKC1125 (0.5 μM) and 5-FU (20 μg/ml). Data are expressed as in Fig. 1**a**. **f** DKC1125 effectively suppresses colorectal liver metastasis, and the combination of DKC1125 with 5-FU exhibits more enhanced therapeutic effect. Experimental hepatic metastasis model was prepared by performing intrasplenic inoculation of stably CT-26/KITENIN-iRFP-expressing cells in syngeneic mice and subsequent splenectomy. For 2 weeks, mice were given intraperitoneal injections of 5-FU once in the entire 2 weeks (100 mg/kg); DKC1125 (10 mg/kg), or vehicle (0.1% DMSO) 3 times/week; or the combination of DKC1125 and 5-FU, respectively. To evaluate metastasis, metastatic tumor growth was calculated as the number of nodules that migrated to the surface of the liver, multiplied by size; this yielded a metastatic score (upper). In addition, total fluorescence emitted from liver nodules expressing iRFP was measured (lower). Metastatic score (left) or total fluorescence (right) are represented as mean ± SEM. An asterisk in a line graph indicates a significant difference between the indicated groups (*p < 0.05, **p < 0.01). **g** Schematic showing how DKC1125 breaks down the functional KITENIN complex and thereby alters the specific cellular context induced by the upregulated complex. Through these mechanisms, DKC1125 exhibits more effective anticancer action in cancer cells expressing higher levels of KITENIN levels. SCC, specific cellular context

DNA-damaging chemotherapeutic reagents such as 5-FU and oxaliplatin, which induce cell cycle arrest and apoptosis, are the main components of combinatorial chemotherapy for CRC patients [38]. Knockdown of KSRP increases the inhibitory effect of 5-FU on CRC cell proliferation [39], and microenvironment-deregulated miRNAs [40, 41] play specific roles in inducing tumor resistance or sensitivity to anticancer drugs [42, 43]. Because the KITENIN-overexpressing CRC cells deregulated certain microRNAs (Table S1 and S2), we examined the effect of DKC1125 on the antiproliferative effects of 5-FU or oxaliplatin. In CRC cell lines, we evaluated the cytotoxicity of various concentrations of DKC1125 combined with 5-FU or oxaliplatin (Additional file 4: Fig. S4). Cell survival was gradually and significantly decreased by increasing doses of DKC1125 at fixed concentrations of 5-FU (Additional file 4: Fig. S4A-C) or oxaliplatin (Additional file 4: Fig. S4D), whereas DKC1125 alone did not affect cell viability in the tested CRC cell lines. The half-maximal inhibitory concentrations (IC_50_) of the drugs alone or in combination with DKC1125, which were obtained from the cell viability curves at the 48 h time point (Additional file 5: Fig. S5), are shown in Fig. 7a. We observed that CRC cells overexpressing KITENIN had a blunted chemotherapeutic response to 5-FU and oxaliplatin (Fig. 7a). Initially, Caco2 cells overexpressing KITENIN had a blunted response to 5-FU (IC_50_, 0.93 μg/ml) and oxaliplatin (IC_50_, 14.2 μM) relative to control cells expressing EV (IC_50_, 0.75 μg/ml and 11.6 μM, respectively); however, combined treatment with DKC1125 effectively and significantly reduced cell viability to a level similar to that of control cells. In all CRC cells used for cell viability assays, combined treatment with DKC1125 with 5-FU or oxaliplatin restored the IC_50_ of 5-FU or oxaliplatin in KITENIN-overexpressing cells and increased the sensitivity to these agents as a function of DKC1125 concentration (Fig. 7a, Additional file 5: Fig. S5). Thus, although DKC1125 itself had little cytotoxicity in 293 T (Additional file 1: Fig. 1) and human intestinal epithelial cells (data not shown), it increased the cytotoxic effect of 5-FU and oxaliplatin by restoring the sensitivity of cells to these agents; combined treatment of 5-FU or oxaliplatin with DKC1125 represented potentiation of the cytotoxic effect of 5-FU or oxaliplatin by DKC1125. These results indicated that treatment with DKC1125 reset the chemosensitivity of 5-FU and oxaliplatin in KITENIN-overexpressing cells, which showed a blunted chemotherapeutic response to 5-FU and oxaliplatin.

Previously, we observed that the functional KITENIN complex mediates resistance of cetuximab in CRC patients with higher KITENIN level [12] and is activated in cetuximab-resistant CRC cells [13]. Hence, we investigated whether co-administration of DKC1125 would affect the action of cetuximab on the invasiveness of HCT116 cells. Cell invasion was further significantly reduced by increasing doses of DKC1125 when doses of cetuximab were fixed (Fig. 7b, left). DKC1125 also restored the IC_50_ of cetuximab for cell invasion by HCT116 cells (Fig. 7b, right). We next tested whether co-administration of DKC1125 would affect the cytotoxicity of combined 5-FU and cetuximab treatment in HCT116 cells. Interestingly, DKC1125 further significantly suppressed cell survival in a gradual manner in the presence of 5-FU and cetuximab (Fig. 7c), a combinatorial regimen currently in wide use for CRC chemotherapy [3]. These in vitro results indicate that DKC1125 represents a new component for use in combination with existing anticancer agents, such as 5-FU/oxaliplatin and cetuximab, to achieve therapeutic enhancement in patients with CRCs expressing higher levels of KITENIN.

### DKC1125 effectively suppresses colorectal liver metastasis, and the combination of DKC1125 with 5-FU exhibits a stronger therapeutic effect

We first tested the anti-tumor effects of DKC1125 in vivo using a syngeneic tumor model in BALB/c mice and the CT-26 murine colon adenocarcinoma cell line. Considering the lipid/water solubility of DKC1125 compound, the maximal dosage of administration in 0.1% DMSO vehicle is 10 mg/kg. Therefore, the initial test dosage was set to 5 mg/kg and was administered every other day for 1 month via intraperitoneal injection from 7 days after cell injection. Average tumor size was significantly larger in the KITENIN group than in the EV group 1 month after injection of tumor cells. DKC1125 had no effect on tumor growth in the EV group; however, tumor growth in the KITENIN group was reduced to a level similar to that in the EV group (Fig. 7d). These results imply that DKC1125 inhibits the progression of CRC triggered by elevated KITENIN expression.

Before investigating the in vivo effect of DKC1125 on distant metastasis of CRC in a mouse model, we tested whether co-administered DKC1125 affects the action of 5-FU on the invasiveness of CT-26 murine colon adenocarcinoma cells. Treatment with DKC1125 also increased the inhibitory effect of 5-FU on the invasion of CT-26 cells (Fig. 7e, left). 5-FU at the dose used in this study (20 μg/ml) had little effect on cell invasiveness. In general, cytotoxic anticancer agents have stronger effects on cell viability rather than cell motility [38]. However, upon co-treatment of 5-FU with DKC1125 (0.5 μM) in CT-26 cells overexpressing KITENIN, cell invasion was a little more decreased (*p* < 0.05) than that of DKC1125 alone. In CT-26 cells overexpressing KITENIN, we observed ~ 55% fewer invading cells (*p* < 0.01) by combined treatment relative to that of 5-FU alone, but this enhanced effect was not observed in CT-26 cells expressing EV (Fig. 7e, right). Because DKC1125 increased the suppressive effect of 5-FU on cell survival and motility in KITENIN-overexpressing CRC cells (Figs. 7a and e), we next investigated the in vivo effect of DKC1125 on distant metastasis of CRC in a mouse model of hepatic metastases using a combinatorial strategy, alone or combination with 5-FU. To create the experimental models, we performed intrasplenic inoculation of stably CT-26/KITENIN-iRFP-expressing cells in syngeneic mice, or of HCT116 cells in nude mice, and the mice were allowed to recover for 2 weeks after subsequent splenectomy. For 2 weeks, mice received intraperitoneal injection of 5-FU once in the entire 2 weeks (100 mg/kg in syngeneic mice; 50 mg/kg in nude mice), or DKC1125 (5, 10 mg/kg) or vehicle 3 times/week, or a combination of 5-FU and DKC1125, respectively. To evaluate metastasis, tumor nodules that migrated to the surface of the liver were counted and multiplied by the size to obtain a metastatic score [23]; metastasis was also evaluated by detecting total fluorescence emitted from tumor nodules expressing iRFP. In the group injected with KITENIN-overexpressing CT-26 cells, DKC1125 alone did not significantly suppress hepatic metastasis relative to 5-FU alone at a dose of 5 mg/kg (Additional file 6: Fig. S6A), the initial setting dosage administered every other day for 1 month in a syngeneic tumor model (Fig. 7d). Next, dosage of DKC1125 was increased to 10 mg/kg, doubling of initial dose, in subsequent experiment. DKC1125 alone at 10 mg/kg clearly suppressed hepatic metastasis relative to 5-FU alone, as assessed by the metastatic score or total fluorescence (Fig. 7f). It is possible that the suppressive effects by DKC1125 are due to the disintegration of the KITENIN complex, which controls cell invasiveness, whereas the effects of 5-FU are due to its cytotoxicity. When DKC1125 was co-administered with 5-FU, the metastatic score or total fluorescence was reduced to a greater extent than by 5-FU or DKC1125 alone (Fig. 7f). As in the in vitro assay, combined treatment had a stronger suppressive effect on distant metastasis due to inhibition of cell invasiveness and restoration of the chemosensitivity of 5-FU. Similar results were obtained using nude mice and HCT116 cells (Additional file 6: Fig. S6B). Again, metastatic liver nodules were suppressed to a greater extent when DKC1125 was administered in combination with 5-FU than when 5-FU was administered alone.

## Discussion

Here, we have introduced several cancer-promoting factors associated with the KITENIN complex and describe how they are altered by a new KSRP-binding compound, DKC1125 (Fig. 7g). DKC1125 binds with KSRP and subsequently releases RACK1 and Dvl2 from KSRP in the KITENIN complex, inducing interaction of RACK1 with Dvl2 or KITENIN and leading to the autophagic degradation of Dvl2 and KITENIN. This destroys the functional KITENIN-KSRP-RACK1-Dvl2 complex and suppresses its oncogenic function. Additionally, DKC1125 increases the expression and maturation of miR-124-3p by KSRP. As a result, miR-124 negatively regulates the KITENIN level. We propose that with the disintegration of the KITENIN complex after DKC1125, the maturation of certain miRNAs such as miR-124 and miR-143 is restored, and these changes lead to the alteration of cellular context induced by the KITENIN complex. These in turn caused recovery of cellular invasiveness and certain microRNAs being affected by elevated KITENIN. The computer docking model of DKC1125-bound KSRP suggested that the maturation of microRNAs by KSRP [25] would be affected by the modified structure of the KH34-domain following insertion of DKC1125 into the binding pocket of the KH4-domain. Thus, DKC1125 disorganized the specific cellular context of the KITENIN complex and had a remarkable effect on cancer cells expressing high levels of KITENIN and RACK1. Therefore, DKC1125 targeted the oncogenic KITENIN complex, decreasing cellular invasiveness and normalizing the expression of specific microRNAs deregulated by elevated KITENIN, such as miRNA-124. Given that DKC1125 increased the suppressive effect of 5-FU on cell survival and motility in KITENIN-overexpressed CRC cells, we investigated the in vivo effect of DKC1125 on distant metastasis of CRC in a mouse colorectal liver metastasis model with combinatorial strategy: either alone or in combination with 5-FU. Alone, DKC1125 and 5-FU each effectively suppressed colorectal liver metastasis to the same degree. However, DKC1125 combined with 5-FU increased the therapeutic effect due to inhibition of cellular invasiveness and restoration of chemosensitivity to 5-FU. Thus, DKC1125 represents a new agent that could be used in combination with existing anticancer therapeutics, such as 5-FU/oxaliplatin and cetuximab, to overcome distant metastasis and chemoresistance in CRC expressing higher levels of KITENIN.

In addition, we have shown that expression of KITENIN is significantly higher in human colon [10, 11], laryngeal [44], oral cavity squamous [45], gastric [46], hepatocellular [47], and glioma [48] tumor tissues than in corresponding normal mucosa. Therefore, given that DKC1125 has no substantial cytotoxicity in cells with lower KITENIN levels, this compound may have more effective anticancer action in cancers with higher KITENIN expression.

Resistance or sensitivity of malignancies to certain pharmacological agents depends not only on the intrinsic traits of cancer cells, but also on the specific tumor microenvironment [49, 50]. In this regard, several miRNAs play critical roles in the interactions between the tumor and microenvironment, and microenvironment-deregulated miRNAs play specific roles in inducing tumor resistance or sensitivity to anticancer drugs [42, 43]. Thus, identification of the miRNAs involved in chemoresistance may provide new therapeutic options or prognostic biomarkers. Currently, 5-FU-containing regimens are a common prescription for treatment of patients with CRC [51], but a large percentage of patients have tumors that are resistant to 5-FU, representing a major barrier to therapy [52]. miRNAs may affect 5-FU resistance or sensitivity by regulating the main signaling pathways involved in CRC, including the PI3K/AKT, Wnt/β-catenin, and Notch signaling pathways [53]. Also, the mechanisms underlying oxaliplatin resistance are complex, but miRNAs such as miR-34a, miR-143, miR-153, miR-27a, miR-218, and miR-520 play essential roles in chemotherapy resistance by targeting various cellular and molecular pathways, e.g., PI3K/Akt/Wnt, EMT, p53, p21, and ATM [54].

In this study, in addition to miR-124-3p, miR-143-3p, and miR-150-5p, we found that several miRNAs were downregulated in KITENIN-overexpressing cells; these miRNAs suppress progression of various cancers including CRC, and their levels were restored after treatment with DKC1125 (Table S3). Downregulation of these miRNAs might activate the PI3K/AKT (due to decreases of miR-34b/c, miR-98, miR-100, miR-101, miR-125a/b, miR-133a, miR-218, miR-486, and miR-622), Wnt/β-catenin (due to decreases of miR-34b/c, miR-98, miR-100, miR-101, miR-124, miR-133a, miR-125b, and miR-140), and Notch signaling pathways (due to a decrease of miR-34b/c, miR-101, miR-124, and miR-140). Indeed, miRNAs responsible for chemosensitivity of 5-FU and oxaliplatin, such as miR-124 and miR-143, were elevated after treatment with DKC1125 (> 40 and > 60%, respectively, Fig. 5a). Thus, we speculate that miRNAs modulated by the KITENIN complex are responsible for the blunted cytotoxic response to 5-FU or oxaliplatin in CRC cells expressing higher levels of KITENIN, and that recovery or upregulation of miRNAs after DKC1125 treatment are responsible for restoring chemosensitivity to 5-FU or oxaliplatin.

Resistance to cetuximab in CRC is mediated by activation of compensatory pathways through modulation of microRNAs to adapt and survive under EGFR inhibition, e.g., through upregulation of miR-199a-5p and miR-375, which target PHLPP1 [55], upregulation of miR-100 and miR-125b via activation of Wnt/β-catenin signaling [56], or downregulation of miR-302a, which targets NFIB and CD44 [57]. Previously, we observed that higher levels of KITENIN are co-expressed in tumor tissues from metastatic CRC patients who exhibit poor responses to cetuximab/chemotherapy [12, 13], and that HCT116 and Caco2 cells expressing higher levels of endogenous KITENIN are more resistant to cetuximab than DLD1 and SW620 cells expressing lower KITENIN [12]. These observations indicated that the KITENIN/ErbB4-c-Jun axis confers resistance to cetuximab in CRC cells, in which the KITENIN axis functions as an unconventional EGFR-independent signal pathway for EGF. In this study, downregulation of miR-124 under higher KITENIN expression (Fig. 5a) was responsible for resistance to cetuximab through upregulation of KITENIN, which is one of the targets of miR-124 and an unconventional downstream effector of EGF. In addition, DKC1125 restored the inhibitory action of cetuximab on cell invasion in HCT116 cells by restoring miR-124 and disintegrating the KITENIN complex. Based on this background, DKC1125 might be used as an alternate combinatorial therapeutic to supplement the limited clinical efficacy of anti-EGFR agents in the subset of CRC patients with KITENIN expression. In this regard, targeting the KITENIN complex represents a promising strategy for overcoming cancer resistance and improving therapeutic outcomes in CRC through precise intervention.

## Conclusion

In summary, our previous and present results indicate that the functional KITENIN complex acts as a platform for promoting cell motility and tumor metastasis, but also for acquisition of resistance to anticancer agents, such as 5-FU and cetuximab. Here, we introduced the KSRP-binding compound DKC1125, which targets the oncogenic KITENIN complex and alters its specific cellular context, and thereby could be used to overcome distant metastasis and chemoresistance in multiple cancers, as well as in CRC expressing higher KITENIN.

## Supplementary Information

**Additional file 1: Supplementary Figure 1.** Identification of DKC1125, a compound that suppresses the KITENIN–AP-1 axis. **a** Diagram of the screen for blockers of AP-1 activity in KITENIN-overexpressing cells. 293 T cells were co-transfected with the AP-1 reporter (1 μg), *Renilla* luciferase (200 ng), KITENIN-V5 (5 μg), and ErbB4-HA (5 μg), and then co-transfected cells (1 × 10^4^) were plated into each well. Forty-eight hours after plating, three wells were treated for 24 h with the same chemical from a small-molecule compound library containing about 6800 species (provided by Korea Chemical Bank). Differences in luciferase activity in the presence or absence of chemicals were examined using the One-Glo system (Promega) or by monitoring cell viability using the Quanti-Max WST-8 Cell Viability Assay Kit. This screening procedure was repeated twice, and about 200 compounds were selected according to the following criteria: reduction of AP-1 reporter over 50% and no substantial cytotoxicity relative to the vehicle-treated group. Next, immunoprecipitation was conducted using Caco2 CRC cells to determine which of the compounds interferes with interaction between KITENIN and ErbB4; 28 were selected, of which 13 compounds that decreased expression of the AP-1 target gene were further selected. Finally, five candidate compounds were selected after confirming suppression of the KITENIN-mediated increase in cell invasion in both Caco2 and MDA-MB231 cells. **b** Inhibition of AP-1 activity in KITENIN-overexpressing 293 T cells by selected compounds from the small-molecule compound library. **c** Suppression by the selected compounds of the KITENIN overexpression-mediated increase in cell invasion in Caco2 CRC cells and MDA-MB231 breast cancer cells. **d** Effects on cell viability of the indicated concentrations of selected compounds in 293 T cells. **e** Silver-stained gel of proteins pulled down using a chemical probe. Proteins pulled down by Affigel alone (AG) or by the DKC1125 chemical probe (Chem-AG) from the HCT116 cell lysate were separated by SDS-PAGE and stained with Coomassie blue. Bands that differed between groups (indicated by arrows) were excised and subjected to proteomic identification using Matrix-assisted laser desorption/ionization time-of-flight mass spectrometry (MALDI-TOF MS). Note the specific band at 75 kDa (indicated by asterisk, *).

**Additional file 2: Supplementary Figure 2.** Binding of DKC1125 with the third and fourth KH-domains of KSRP. Isothermal titration calorimetry (ITC) was performed to support the binding of DKC1125 to the KH-domains of KSRP, which are known RNA-binding targets within KSRP. The results confirmed that DKC1125 binds with the third and fourth KH-domains of KSRP.

**Additional file 3: Supplementary Figure 3.** Characteristics of Dvl and RACK1 in the functional KITENIN complex, and the influence of DKC1125 treatment. **a** Dvl2 binds with KSRP via the PDZ domain. Three deletion mutants of HA-Dvl were expressed in Caco2 cells, and cell lysates were immunoprecipitated with anti-KSRP antibody and immunoblotted with anti-HA antibody to detect the interaction between endogenous KSRP and HA-tagging Dvl. Each Dvl deletion mutant that bound KSRP is indicated by an arrowhead. **b** KSRP interacts with RACK1 via the KH12-domain and with KITENIN via the C-terminal domain. To identify the RACK1-binding or KITENIN-binding site of KSRP, co-IPs were carried out in Caco2 cells after transfection of various myc-tagged deletion mutants of KSRP [full-length KSRP-myc (WT), ΔKH12-KSRP-myc (Δ12), Δ34KH-KSRP-myc (Δ34), and ΔC-term-KSRP-myc (ΔC)] and GFP-tagged RACK1 or V5-tagged KITENIN (middle panel). **c** Whole-cell lysates of several deletion mutants of KSRP co-expressed in Caco2 cells with RACK1-GFP. IP data using these cell lysates are represented in Fig. 6**b**. **d** DKC1125 also promotes degradation of Dvl2 through induction of RACK1. Levels of RACK1 and Dvl2 were examined in several CRC cell lines after DKC1125 treatment (0.5 μM). **e** DKC1125 accelerates autophagic degradation of KITENIN under RACK1 expression. Caco2 cells were transfected with empty vector (EV) or RACK1-GFP. They were initially pretreated with vehicle, MG132 (MG, 10 μM), bafilomycin A1 (A1, 100 nM), or chloroquine (CQ, 10 μM), and later treated with vehicle or DKC1125 (0.5 μM). The level of KITENIN was determined by immunoblot analysis (right panel).

**Additional file 4: Supplementary Figure 4.** Differences in cell survival after treatment with 5-FU or oxaliplatin in the presence or absence of DKC1125 in CRC cells overexpressing KITENIN. Various CRC cells (CT-26, Caco2, HCT116, and HT29) were seeded at 5 × 10^3^ cells/well on 96-well plates, and the cytotoxicity of the indicated concentrations of 5-fluorouracil (5-FU, 0.02, 0.1, 0.5 μg/ml, **a**-**c**) or oxaliplatin (oxa, 1, 3, 10 μM, **d**) combined with DKC1125 (#25, 0.5, 1.5, 4.5 μM) was examined by tetrazolium salt (WST-8) assay 72 h after seeding. Data shown in line graphs (mean ± SEM, *n* = 3) were compared among groups at three time points (24, 48, and 72 h). The optical density (O.D.) values of each time point (24, 48, 72 h) were recalculated using the criterion that the O.D. value of 24 h with no treatment (5-FU and DKC1125) is set to 1.0. The asterisk indicates a significant difference between the indicated groups at each time point (^*^P < 0.05; ^**^P < 0.01). In CT-26 cells, the asterisk indicates at one time point (72 h). Cell viability was further gradually suppressed by increasing doses of DKC1125 at fixed concentrations of 5-fluorouracil (**a**-**c**), which were treated for three time points (24, 48, and 72 h), or oxaliplatin (**d**), which were treated for 48 h.

**Additional file 5: Supplementary Figure 5.** Differences in cell cytotoxicity at 48 h after treatment with 5-FU or oxaliplatin in the presence or absence of DKC1125 in CRC cells overexpressing KITENIN. To obtain the 48 h IC_50_ values of 5-FU or oxaliplatin and for statistical validation, the cell viability test using various CRC cells (Caco2, CT-26, HCT116, and HT29) was repeated at the 48 h time point three times as in Fig. S4. The cytotoxicity of the indicated concentrations of 5-fluorouracil (5-FU, 0.02, 0.1, 0.5 μg/ml, **a**-**d**) or oxaliplatin (oxa, 1, 3, 10 μM, **e, f**) combined with DKC1125 (#25, 0.5, 1.5, 4.5 μM), which were treated for 48 h, was examined by tetrazolium salt (WST-8) assay. The O.D. values of CRC cells overexpressing KITENIN are recalculated using the criterion that the O.D. value of CRC cells expressing empty vector (EV) with no treatment (5-FU and DKC1125) is set to 1.0. Data are expressed as in Fig. S4. The 48 h IC_50_ values in Fig. 7**a** were calculated from these cell viability data.

**Additional file 6: Supplementary Figure 6.** The combination of DKC1125 with 5-FU exerts a stronger therapeutic effect on colorectal liver metastasis than 5-FU alone. **a** Suppressive effect of DKC1125 (5 mg/kg) on hepatic metastasis of syngeneic mice. In the group injected with KITENIN-overexpressing CT-26 cells, DKC1125 (5 mg/kg, an initial dosage administered every other day for 1 month in a syngeneic tumor model, Fig. 7**d**) did not significantly suppress hepatic metastasis relative to 5-FU alone. **b** Liver metastasis was suppressed more by combination of DKC1125 with 5-FU in nude mice than 5-FU or DKC1125 alone. The experimental hepatic metastasis model was prepared by intrasplenic inoculation of stably CT-26/KITENIN cells into syngeneic mice (**a**), or of HCT116 cells into nude mice (**b**), followed by splenectomy. For 2 weeks, mice were given intraperitoneal injection of 5-FU once in the entire 2 weeks (100 mg/kg in syngeneic mice; 50 mg/kg in nude mice); DKC1125 (5 mg/kg in syngeneic and in nude mice) or vehicle (0.1% DMSO) 3 times/week; or a combination of DKC1125 and 5-FU. For evaluation of metastasis, metastatic tumor growth was counted as nodules that migrated to the surface of the liver and multiplied by size to obtain a metastatic score. Metastatic scores are represented as means ± SEM. An asterisk (*p < 0.05, **p < 0.01) indicates a significant difference relative to the vehicle-treated group (**a**) or a significant difference between the indicated groups (**b**).

**Additional file 7: Supplementary Table 1.** Changes in microRNAs associated with the cancer pathway under KITENIN overexpression and the effects of DKC1125 on these changes. The expression of microRNAs were examined using the Pathway-focused miScript miRNA PCR array. Data were represented as mean ± SEM (*n* = 3). C, empty vector-transfected CRC cells in control group; CK, CRC cells overexpressing KITENIN in control group; C-V, empty vector-transfected CRC cells treated with vehicle (0.1% DMSO) alone; CK-V, CRC cells overexpressing KITENIN treated with vehicle alone; C-DKC, empty vector-transfected CRC cells treated with DKC1125 (0.5 μM); CK-DKC, CRC cells overexpressing KITENIN treated with DKC1125.

**Additional file 8: Supplementary Table 2.** Changes in tumor suppressor microRNAs under KITENIN overexpression, and the effects of DKC1125 on these changes. Based on the results of suppressor miRNA in KITENIN-overexpressed CRC cells (Supplementary Table 1), suppressor miRNAs whose levels changed in CRC cells expressing high KITENIN and following treatment with DKC1125 were investigated using the Pathway-focused miScript miRNA PCR array. Data were represented as mean ± SEM (*n* = 3).

**Additional file 9: Supplementary Table 3.** Several suppressor microRNAs whose levels decreased under KITENIN overexpression but recovered after DKC1125. Suppressor miRNAs whose levels were decreased to a greater extent in CRC cells expressing high KITENIN than in parental cells (> 25%) and restored after treatment with DKC1125 (Supplementary Table 1 and 2) are summarized. Data were represented as mean ± SEM (*n* = 3). In addition to miR-124-3p, miR-143-3p, and miR-150-5p, several other suppressor miRNAs are shown: miR-100-5p, miR-125a-5p, miR-125b-5p, miR-140-5p, miR-218-5p, miR-34c-5p, miR-98-5p, miR-101-3p, miR-133a-3p, miR-216b-5p, miR-34b-3p, miR-486-5p, miR-502-5p, and miR-622.

## Data Availability

All the data generated or analyzed during this study are included in this published article and its supplementary files.

## Abbreviations

KITENIN: KAI1 C-terminal interacting tetraspanin
CRC: Colorectal cancer
DKC1125: Disintegrator of KITENIN Complex #1125
KSRP: KH-type splicing regulatory protein
KITENIN-GOF: KITENIN gain of function
5-FU: 5-Fluorouracil
Dvl: Dishevelled
EV: Empty vector
miR: miRNA, microRNA
RACK1: Receptor for activated C-kinase 1
IC50: Half-maximal inhibitory concentration
ITC: Isothermal titration calorimetry
SCC: Specific cellular context
