# Supplementary figures and images for: A new KSRP-binding compound suppresses distant metastasis of colorectal cancer by targeting the oncogenic KITENIN complex
Source: Mol Cancer. 2021 May 26;20:78. doi: 10.1186/s12943-021-01368-w (PMC8152081; doi:10.1186/s12943-021-01368-w)

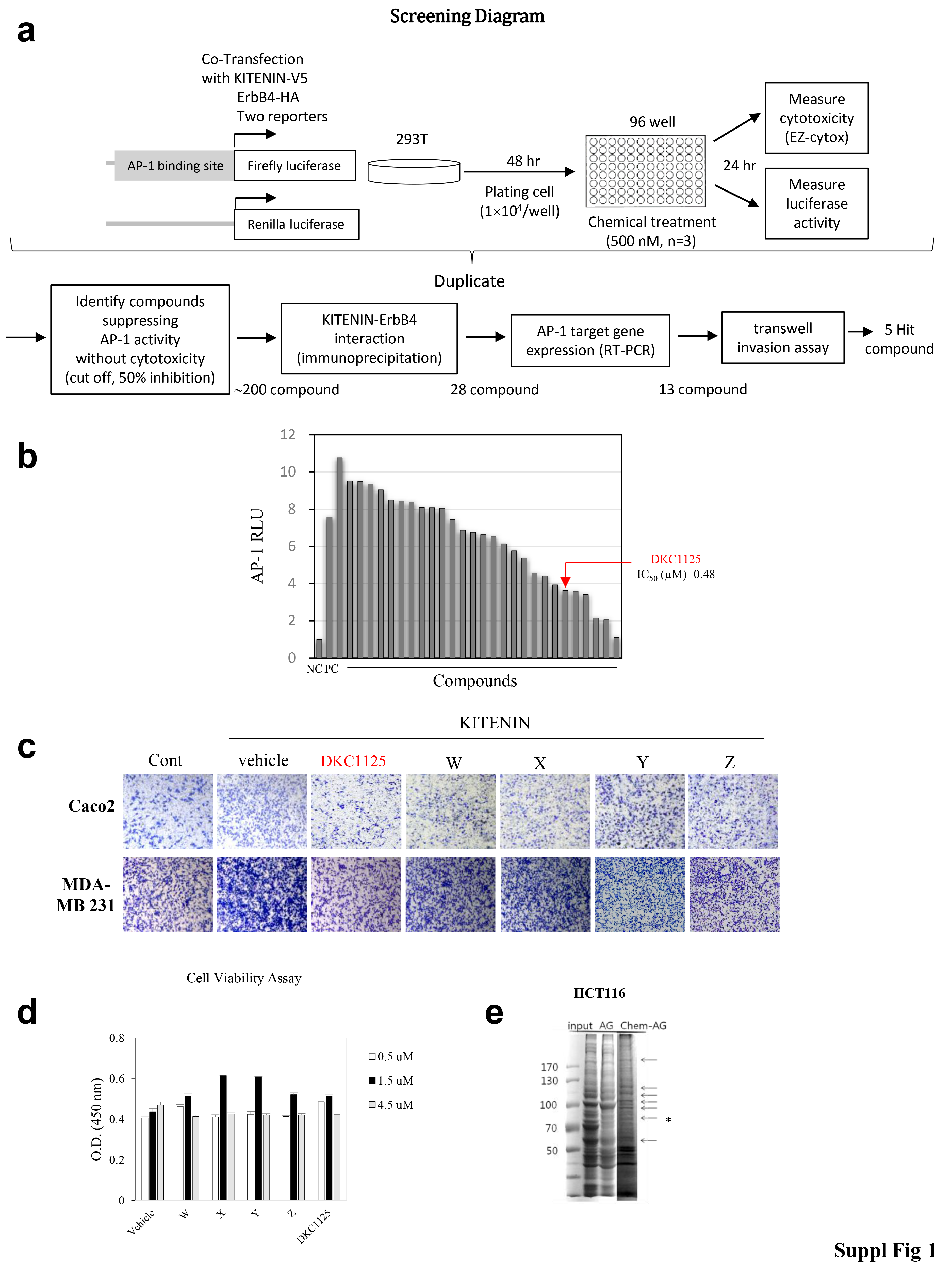

Supplement: Supplementary file 1 — Additional file 1: Supplementary Figure 1. Identification of DKC1125, a compound that suppresses the KITENIN–AP-1 axis. a Diagram of the screen for blockers of AP-1 activity in KITENIN-overexpressing cells. 293 T cells were co-transfected with the AP-1 reporter (1 μg), Renilla luciferase (200 ng), KITENIN-V5 (5 μg), and ErbB4-HA (5 μg), and then co-transfected cells (1 × 10^4^) were plated into each well. Forty-eight hours after plating, three wells were treated for 24 h with the same chemical from a small-molecule compound library containing about 6800 species (provided by Korea Chemical Bank). Differences in luciferase activity in the presence or absence of chemicals were examined using the One-Glo system (Promega) or by monitoring cell viability using the Quanti-Max WST-8 Cell Viability Assay Kit. This screening procedure was repeated twice, and about 200 compounds were selected according to the following criteria: reduction of AP-1 reporter over 50% and no substantial cytotoxicity relative to the vehicle-treated group. Next, immunoprecipitation was conducted using Caco2 CRC cells to determine which of the compounds interferes with interaction between KITENIN and ErbB4; 28 were selected, of which 13 compounds that decreased expression of the AP-1 target gene were further selected. Finally, five candidate compounds were selected after confirming suppression of the KITENIN-mediated increase in cell invasion in both Caco2 and MDA-MB231 cells. b Inhibition of AP-1 activity in KITENIN-overexpressing 293 T cells by selected compounds from the small-molecule compound library. c Suppression by the selected compounds of the KITENIN overexpression-mediated increase in cell invasion in Caco2 CRC cells and MDA-MB231 breast cancer cells. d Effects on cell viability of the indicated concentrations of selected compounds in 293 T cells. e Silver-stained gel of proteins pulled down using a chemical probe. Proteins pulled down by Affigel alone (AG) or by the DKC1125 chemical probe ( [file 12943_2021_1368_MOESM1_ESM.tif]

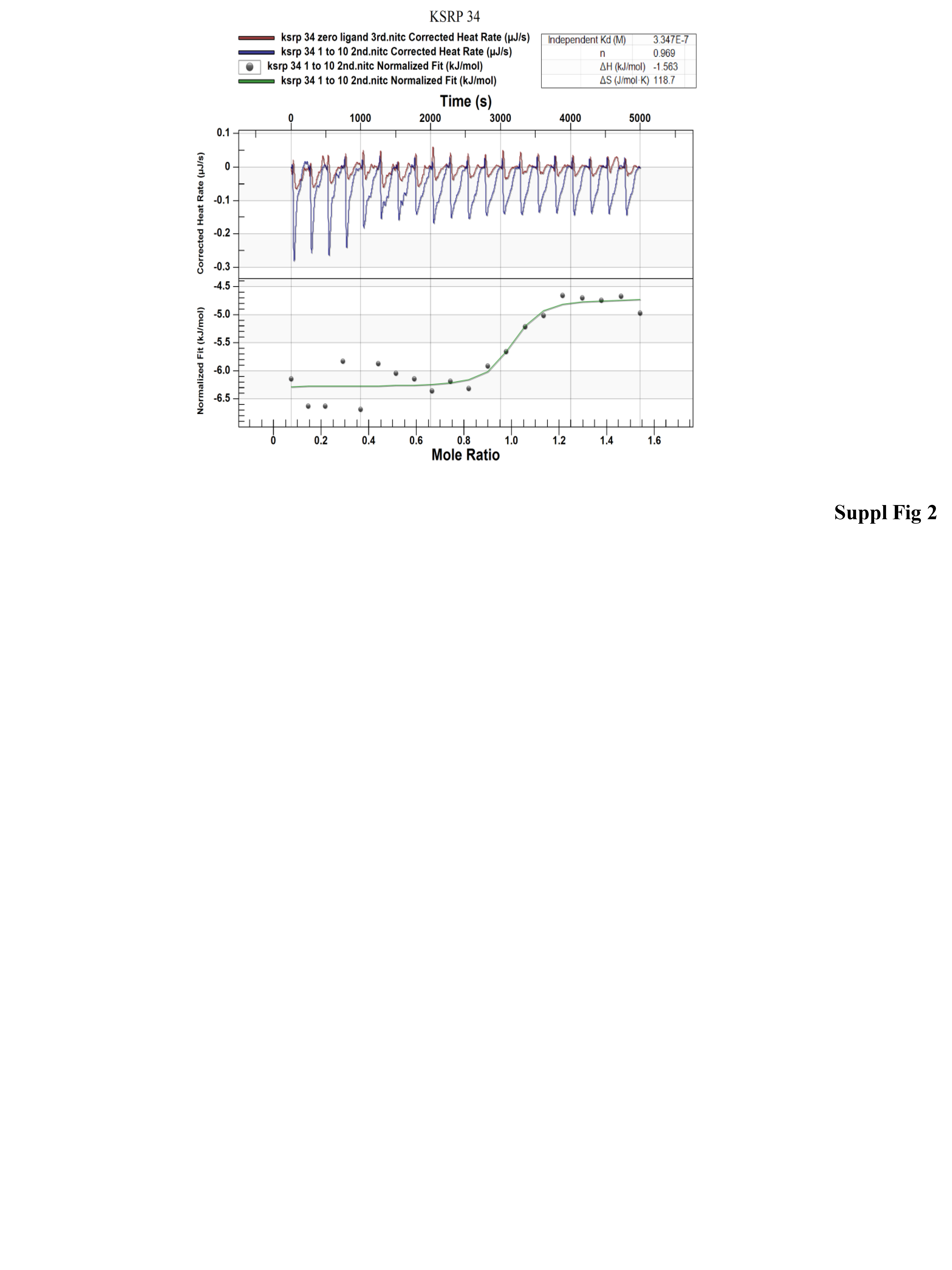

Supplement: Supplementary file 2 — Additional file 2: Supplementary Figure 2. Binding of DKC1125 with the third and fourth KH-domains of KSRP. Isothermal titration calorimetry (ITC) was performed to support the binding of DKC1125 to the KH-domains of KSRP, which are known RNA-binding targets within KSRP. The results confirmed that DKC1125 binds with the third and fourth KH-domains of KSRP. [file 12943_2021_1368_MOESM2_ESM.tif]

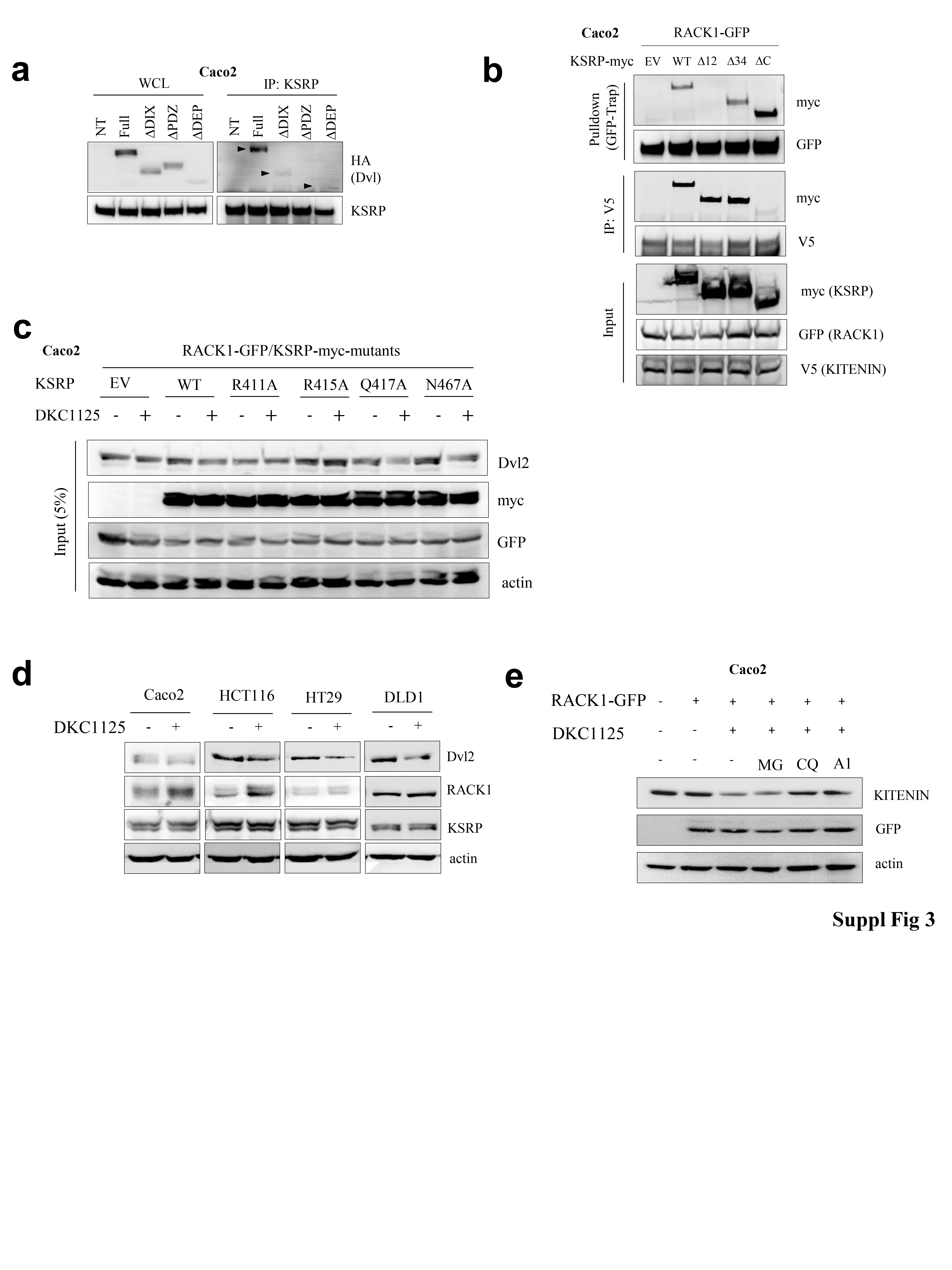

Supplement: Supplementary file 3 — Additional file 3: Supplementary Figure 3. Characteristics of Dvl and RACK1 in the functional KITENIN complex, and the influence of DKC1125 treatment. a Dvl2 binds with KSRP via the PDZ domain. Three deletion mutants of HA-Dvl were expressed in Caco2 cells, and cell lysates were immunoprecipitated with anti-KSRP antibody and immunoblotted with anti-HA antibody to detect the interaction between endogenous KSRP and HA-tagging Dvl. Each Dvl deletion mutant that bound KSRP is indicated by an arrowhead. b KSRP interacts with RACK1 via the KH12-domain and with KITENIN via the C-terminal domain. To identify the RACK1-binding or KITENIN-binding site of KSRP, co-IPs were carried out in Caco2 cells after transfection of various myc-tagged deletion mutants of KSRP [full-length KSRP-myc (WT), ΔKH12-KSRP-myc (Δ12), Δ34KH-KSRP-myc (Δ34), and ΔC-term-KSRP-myc (ΔC)] and GFP-tagged RACK1 or V5-tagged KITENIN (middle panel). c Whole-cell lysates of several deletion mutants of KSRP co-expressed in Caco2 cells with RACK1-GFP. IP data using these cell lysates are represented in Fig. 6b. d DKC1125 also promotes degradation of Dvl2 through induction of RACK1. Levels of RACK1 and Dvl2 were examined in several CRC cell lines after DKC1125 treatment (0.5 μM). e DKC1125 accelerates autophagic degradation of KITENIN under RACK1 expression. Caco2 cells were transfected with empty vector (EV) or RACK1-GFP. They were initially pretreated with vehicle, MG132 (MG, 10 μM), bafilomycin A1 (A1, 100 nM), or chloroquine (CQ, 10 μM), and later treated with vehicle or DKC1125 (0.5 μM). The level of KITENIN was determined by immunoblot analysis (right panel). [file 12943_2021_1368_MOESM3_ESM.tif]

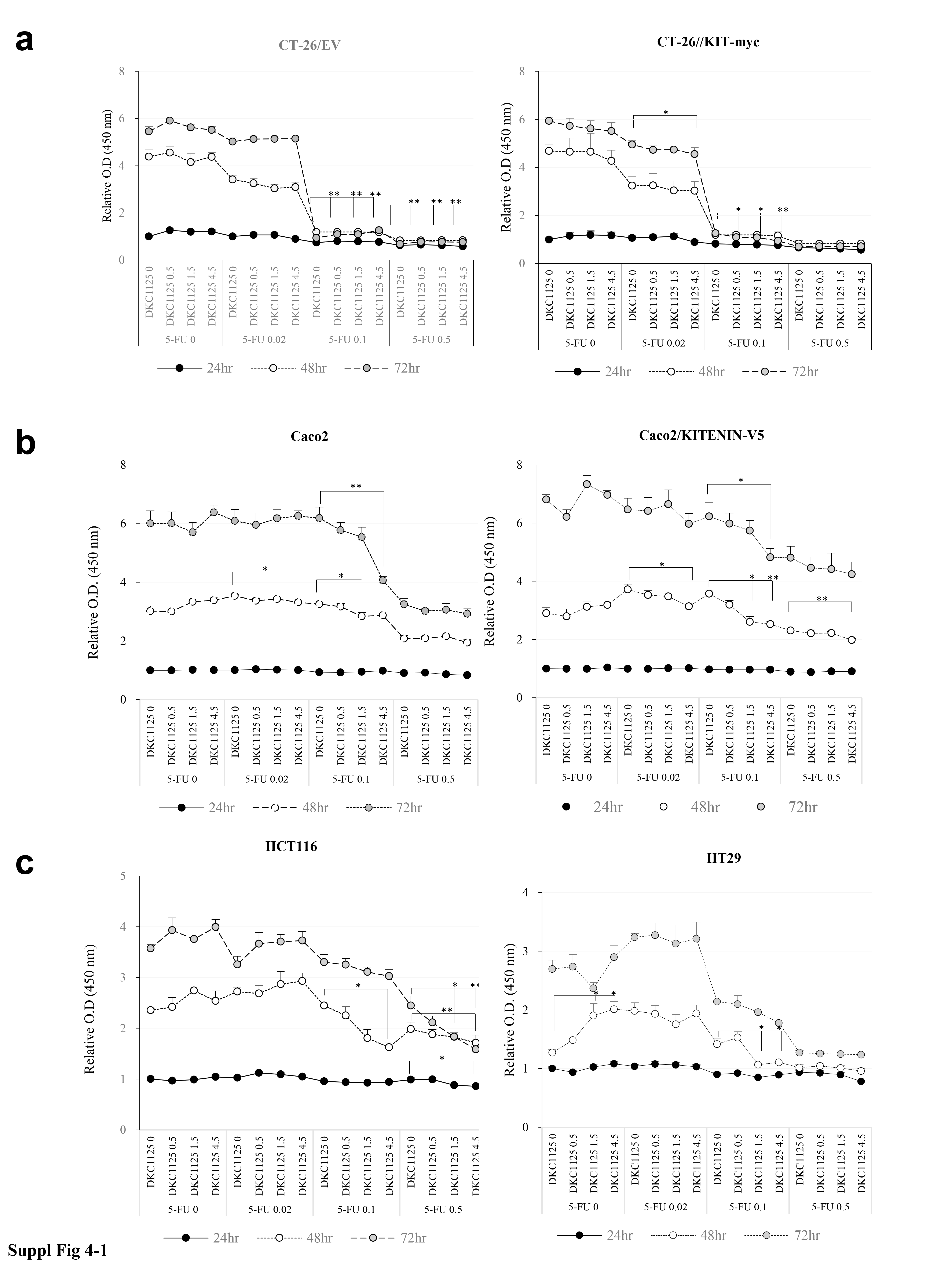

Supplement: Supplementary file 4 — Additional file 4: Supplementary Figure 4. Differences in cell survival after treatment with 5-FU or oxaliplatin in the presence or absence of DKC1125 in CRC cells overexpressing KITENIN. Various CRC cells (CT-26, Caco2, HCT116, and HT29) were seeded at 5 × 10^3^ cells/well on 96-well plates, and the cytotoxicity of the indicated concentrations of 5-fluorouracil (5-FU, 0.02, 0.1, 0.5 μg/ml, a-c) or oxaliplatin (oxa, 1, 3, 10 μM, d) combined with DKC1125 (#25, 0.5, 1.5, 4.5 μM) was examined by tetrazolium salt (WST-8) assay 72 h after seeding. Data shown in line graphs (mean ± SEM, n = 3) were compared among groups at three time points (24, 48, and 72 h). The optical density (O.D.) values of each time point (24, 48, 72 h) were recalculated using the criterion that the O.D. value of 24 h with no treatment (5-FU and DKC1125) is set to 1.0. The asterisk indicates a significant difference between the indicated groups at each time point (*P < 0.05; **P < 0.01). In CT-26 cells, the asterisk indicates at one time point (72 h). Cell viability was further gradually suppressed by increasing doses of DKC1125 at fixed concentrations of 5-fluorouracil (a-c), which were treated for three time points (24, 48, and 72 h), or oxaliplatin (d), which were treated for 48 h. [file 12943_2021_1368_MOESM4_ESM.zip › Suppl Fig 4-1--MOLC-D-21-00356-R2_ESM.tif]

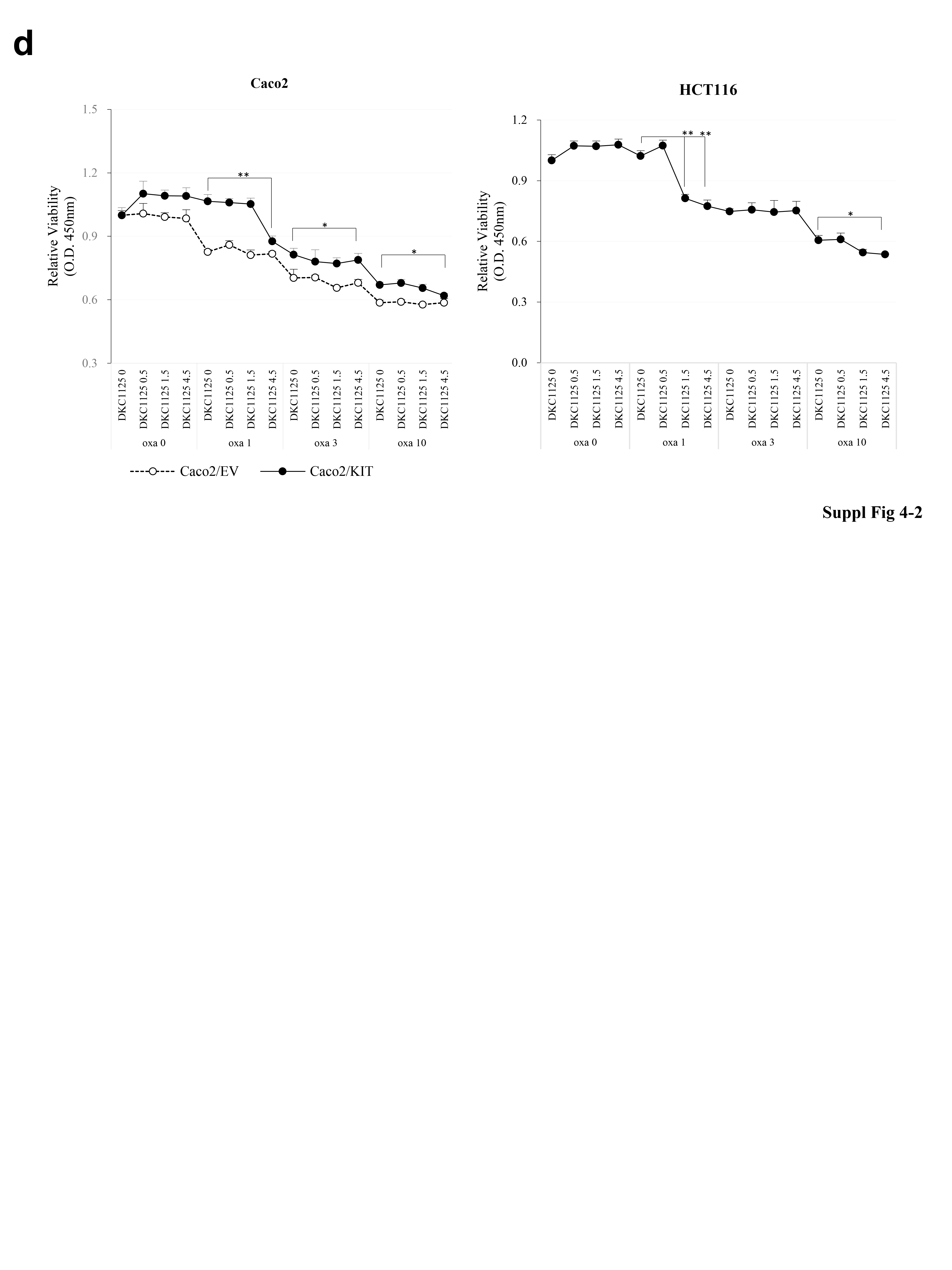

Supplement: Supplementary file 4 — Additional file 4: Supplementary Figure 4. Differences in cell survival after treatment with 5-FU or oxaliplatin in the presence or absence of DKC1125 in CRC cells overexpressing KITENIN. Various CRC cells (CT-26, Caco2, HCT116, and HT29) were seeded at 5 × 10^3^ cells/well on 96-well plates, and the cytotoxicity of the indicated concentrations of 5-fluorouracil (5-FU, 0.02, 0.1, 0.5 μg/ml, a-c) or oxaliplatin (oxa, 1, 3, 10 μM, d) combined with DKC1125 (#25, 0.5, 1.5, 4.5 μM) was examined by tetrazolium salt (WST-8) assay 72 h after seeding. Data shown in line graphs (mean ± SEM, n = 3) were compared among groups at three time points (24, 48, and 72 h). The optical density (O.D.) values of each time point (24, 48, 72 h) were recalculated using the criterion that the O.D. value of 24 h with no treatment (5-FU and DKC1125) is set to 1.0. The asterisk indicates a significant difference between the indicated groups at each time point (*P < 0.05; **P < 0.01). In CT-26 cells, the asterisk indicates at one time point (72 h). Cell viability was further gradually suppressed by increasing doses of DKC1125 at fixed concentrations of 5-fluorouracil (a-c), which were treated for three time points (24, 48, and 72 h), or oxaliplatin (d), which were treated for 48 h. [file 12943_2021_1368_MOESM4_ESM.zip › Suppl Fig 4-2-MOLC-D-21-00356-R2_ESM.tif]

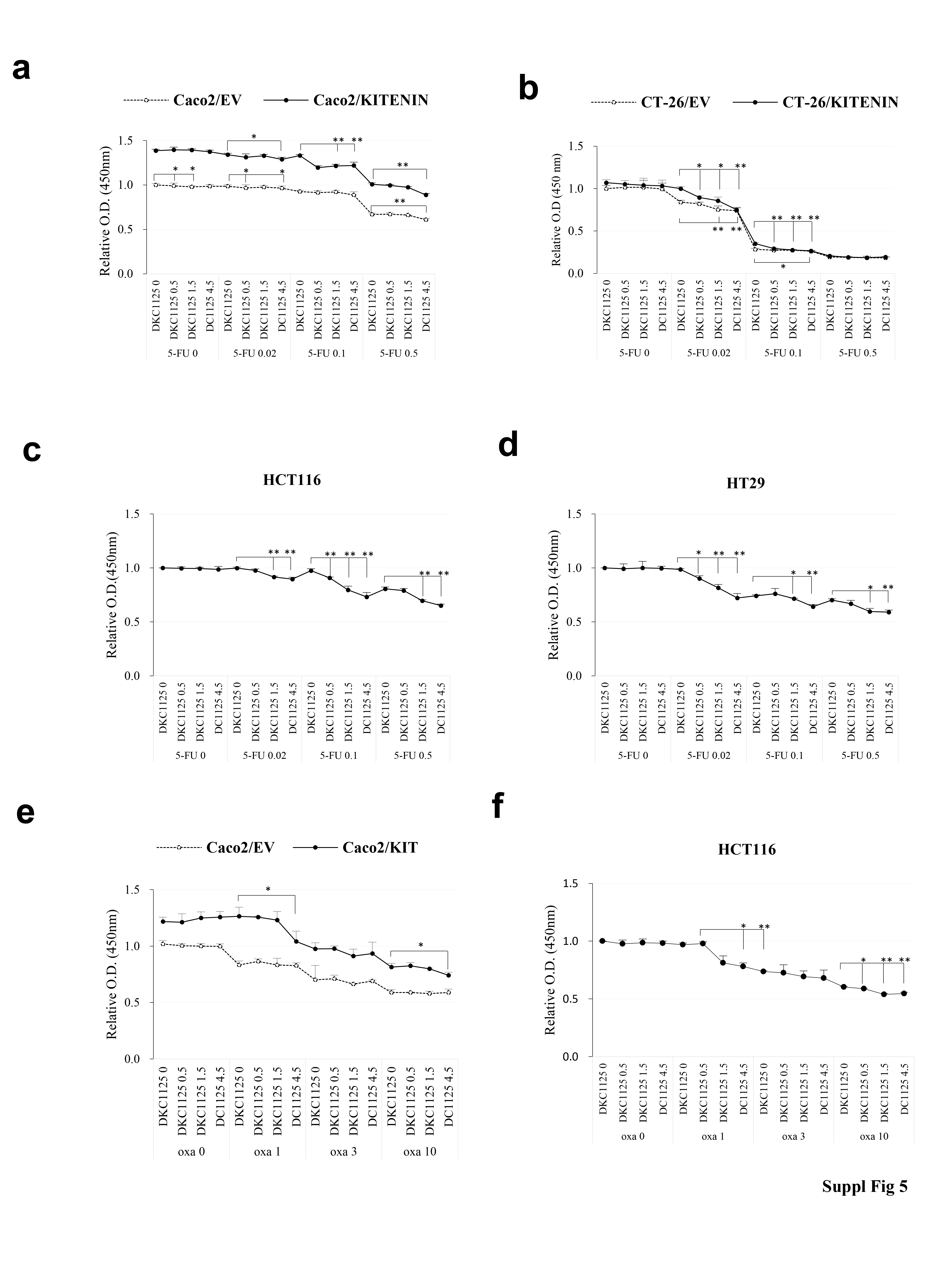

Supplement: Supplementary file 5 — Additional file 5: Supplementary Figure 5. Differences in cell cytotoxicity at 48 h after treatment with 5-FU or oxaliplatin in the presence or absence of DKC1125 in CRC cells overexpressing KITENIN. To obtain the 48 h IC50 values of 5-FU or oxaliplatin and for statistical validation, the cell viability test using various CRC cells (Caco2, CT-26, HCT116, and HT29) was repeated at the 48 h time point three times as in Fig. S4. The cytotoxicity of the indicated concentrations of 5-fluorouracil (5-FU, 0.02, 0.1, 0.5 μg/ml, a-d) or oxaliplatin (oxa, 1, 3, 10 μM, e, f) combined with DKC1125 (#25, 0.5, 1.5, 4.5 μM), which were treated for 48 h, was examined by tetrazolium salt (WST-8) assay. The O.D. values of CRC cells overexpressing KITENIN are recalculated using the criterion that the O.D. value of CRC cells expressing empty vector (EV) with no treatment (5-FU and DKC1125) is set to 1.0. Data are expressed as in Fig. S4. The 48 h IC50 values in Fig. 7a were calculated from these cell viability data. [file 12943_2021_1368_MOESM5_ESM.tif]

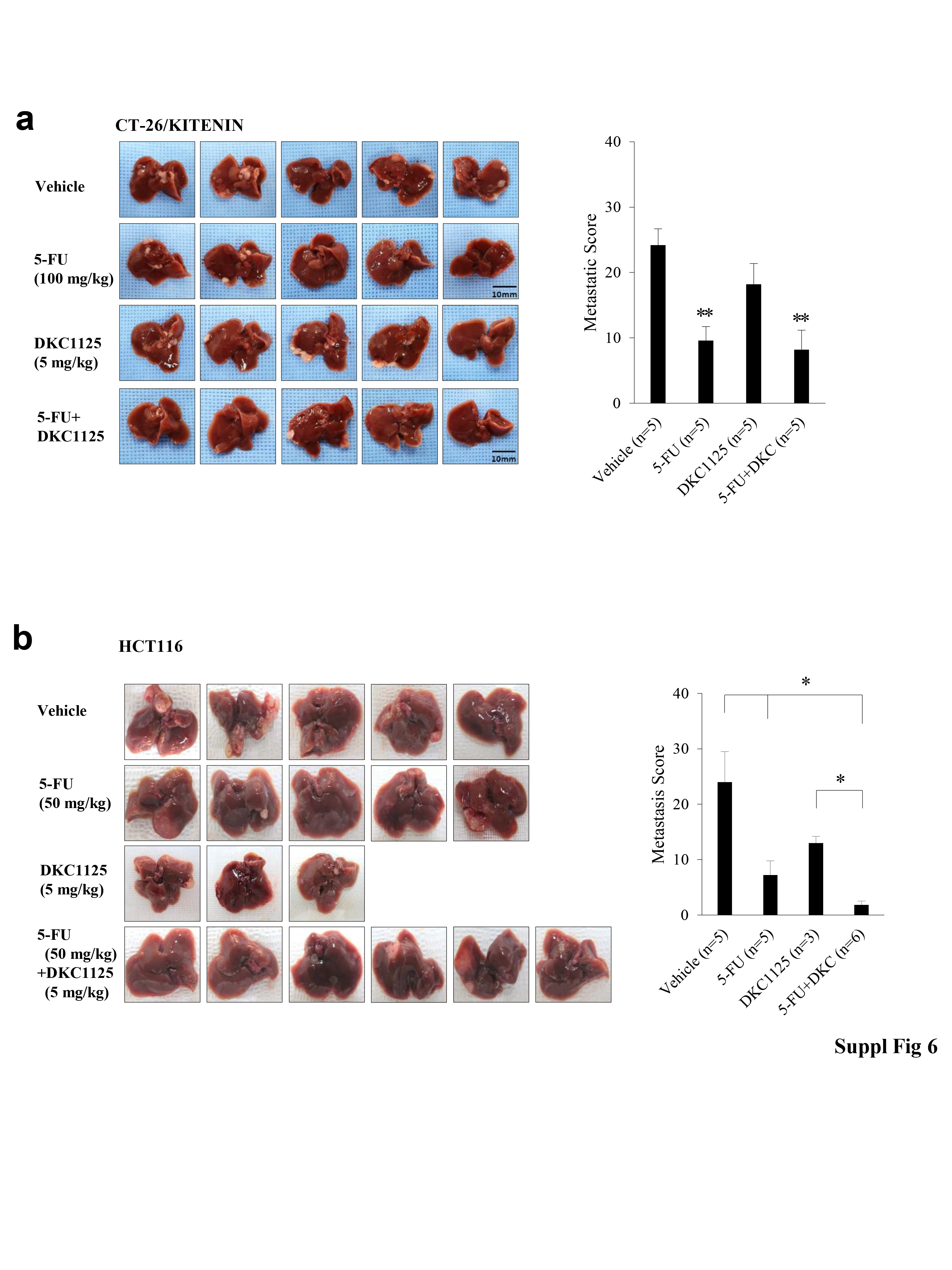

Supplement: Supplementary file 6 — Additional file 6: Supplementary Figure 6. The combination of DKC1125 with 5-FU exerts a stronger therapeutic effect on colorectal liver metastasis than 5-FU alone. a Suppressive effect of DKC1125 (5 mg/kg) on hepatic metastasis of syngeneic mice. In the group injected with KITENIN-overexpressing CT-26 cells, DKC1125 (5 mg/kg, an initial dosage administered every other day for 1 month in a syngeneic tumor model, Fig. 7d) did not significantly suppress hepatic metastasis relative to 5-FU alone. b Liver metastasis was suppressed more by combination of DKC1125 with 5-FU in nude mice than 5-FU or DKC1125 alone. The experimental hepatic metastasis model was prepared by intrasplenic inoculation of stably CT-26/KITENIN cells into syngeneic mice (a), or of HCT116 cells into nude mice (b), followed by splenectomy. For 2 weeks, mice were given intraperitoneal injection of 5-FU once in the entire 2 weeks (100 mg/kg in syngeneic mice; 50 mg/kg in nude mice); DKC1125 (5 mg/kg in syngeneic and in nude mice) or vehicle (0.1% DMSO) 3 times/week; or a combination of DKC1125 and 5-FU. For evaluation of metastasis, metastatic tumor growth was counted as nodules that migrated to the surface of the liver and multiplied by size to obtain a metastatic score. Metastatic scores are represented as means ± SEM. An asterisk (*p < 0.05, **p < 0.01) indicates a significant difference relative to the vehicle-treated group (a) or a significant difference between the indicated groups (b). [file 12943_2021_1368_MOESM6_ESM.tif]
