# Supplementary material for: A new KSRP-binding compound suppresses distant metastasis of colorectal cancer by targeting the oncogenic KITENIN complex
Source: Mol Cancer. 2021 May 26;20:78. doi: 10.1186/s12943-021-01368-w (PMC8152081; doi:10.1186/s12943-021-01368-w)
Supplement: Supplementary file 7 — Additional file 7: Supplementary Table 1. Changes in microRNAs associated with the cancer pathway under KITENIN overexpression and the effects of DKC1125 on these changes. The expression of microRNAs were examined using the Pathway-focused miScript miRNA PCR array. Data were represented as mean ± SEM (n = 3). C, empty vector-transfected CRC cells in control group; CK, CRC cells overexpressing KITENIN in control group; C-V, empty vector-transfected CRC cells treated with vehicle (0.1% DMSO) alone; CK-V, CRC cells overexpressing KITENIN treated with vehicle alone; C-DKC, empty vector-transfected CRC cells treated with DKC1125 (0.5 μM); CK-DKC, CRC cells overexpressing KITENIN treated with DKC1125. [file 12943_2021_1368_MOESM7_ESM.pptx]

## Slide 1
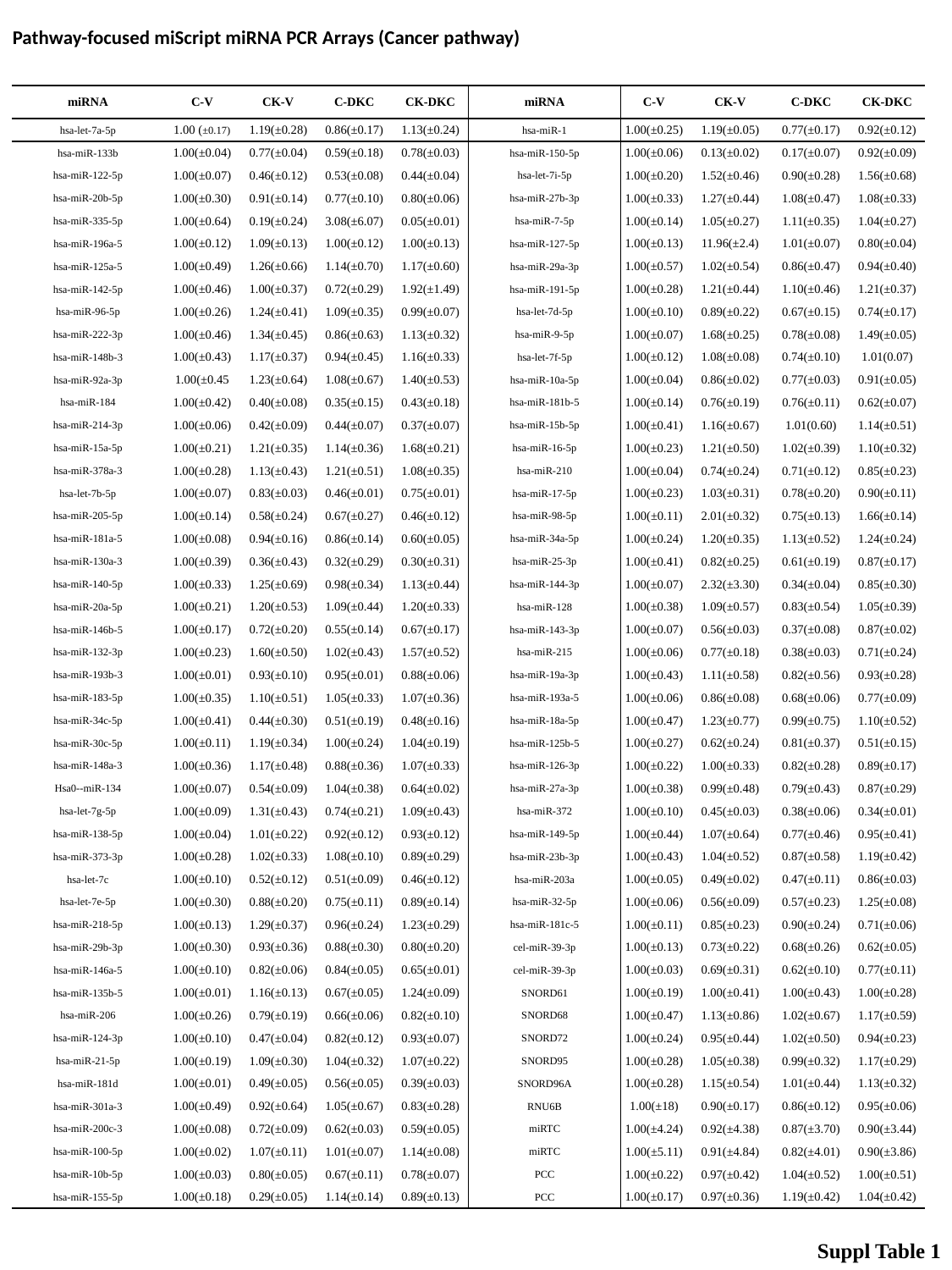

Pathway-focused miScript miRNA PCR Arrays (Cancer pathway)
| miRNA | C-V | CK-V | C-DKC | CK-DKC | miRNA | C-V | CK-V | C-DKC | CK-DKC |
| --- | --- | --- | --- | --- | --- | --- | --- | --- | --- |
| hsa-let-7a-5p | 1.00 (±0.17) | 1.19(±0.28) | 0.86(±0.17) | 1.13(±0.24) | hsa-miR-1 | 1.00(±0.25) | 1.19(±0.05) | 0.77(±0.17) | 0.92(±0.12) |
| hsa-miR-133b | 1.00(±0.04) | 0.77(±0.04) | 0.59(±0.18) | 0.78(±0.03) | hsa-miR-150-5p | 1.00(±0.06) | 0.13(±0.02) | 0.17(±0.07) | 0.92(±0.09) |
| hsa-miR-122-5p | 1.00(±0.07) | 0.46(±0.12) | 0.53(±0.08) | 0.44(±0.04) | hsa-let-7i-5p | 1.00(±0.20) | 1.52(±0.46) | 0.90(±0.28) | 1.56(±0.68) |
| hsa-miR-20b-5p | 1.00(±0.30) | 0.91(±0.14) | 0.77(±0.10) | 0.80(±0.06) | hsa-miR-27b-3p | 1.00(±0.33) | 1.27(±0.44) | 1.08(±0.47) | 1.08(±0.33) |
| hsa-miR-335-5p | 1.00(±0.64) | 0.19(±0.24) | 3.08(±6.07) | 0.05(±0.01) | hsa-miR-7-5p | 1.00(±0.14) | 1.05(±0.27) | 1.11(±0.35) | 1.04(±0.27) |
| hsa-miR-196a-5 | 1.00(±0.12) | 1.09(±0.13) | 1.00(±0.12) | 1.00(±0.13) | hsa-miR-127-5p | 1.00(±0.13) | 11.96(±2.4) | 1.01(±0.07) | 0.80(±0.04) |
| hsa-miR-125a-5 | 1.00(±0.49) | 1.26(±0.66) | 1.14(±0.70) | 1.17(±0.60) | hsa-miR-29a-3p | 1.00(±0.57) | 1.02(±0.54) | 0.86(±0.47) | 0.94(±0.40) |
| hsa-miR-142-5p | 1.00(±0.46) | 1.00(±0.37) | 0.72(±0.29) | 1.92(±1.49) | hsa-miR-191-5p | 1.00(±0.28) | 1.21(±0.44) | 1.10(±0.46) | 1.21(±0.37) |
| hsa-miR-96-5p | 1.00(±0.26) | 1.24(±0.41) | 1.09(±0.35) | 0.99(±0.07) | hsa-let-7d-5p | 1.00(±0.10) | 0.89(±0.22) | 0.67(±0.15) | 0.74(±0.17) |
| hsa-miR-222-3p | 1.00(±0.46) | 1.34(±0.45) | 0.86(±0.63) | 1.13(±0.32) | hsa-miR-9-5p | 1.00(±0.07) | 1.68(±0.25) | 0.78(±0.08) | 1.49(±0.05) |
| hsa-miR-148b-3 | 1.00(±0.43) | 1.17(±0.37) | 0.94(±0.45) | 1.16(±0.33) | hsa-let-7f-5p | 1.00(±0.12) | 1.08(±0.08) | 0.74(±0.10) | 1.01(0.07) |
| hsa-miR-92a-3p | 1.00(±0.45 | 1.23(±0.64) | 1.08(±0.67) | 1.40(±0.53) | hsa-miR-10a-5p | 1.00(±0.04) | 0.86(±0.02) | 0.77(±0.03) | 0.91(±0.05) |
| hsa-miR-184 | 1.00(±0.42) | 0.40(±0.08) | 0.35(±0.15) | 0.43(±0.18) | hsa-miR-181b-5 | 1.00(±0.14) | 0.76(±0.19) | 0.76(±0.11) | 0.62(±0.07) |
| hsa-miR-214-3p | 1.00(±0.06) | 0.42(±0.09) | 0.44(±0.07) | 0.37(±0.07) | hsa-miR-15b-5p | 1.00(±0.41) | 1.16(±0.67) | 1.01(0.60) | 1.14(±0.51) |
| hsa-miR-15a-5p | 1.00(±0.21) | 1.21(±0.35) | 1.14(±0.36) | 1.68(±0.21) | hsa-miR-16-5p | 1.00(±0.23) | 1.21(±0.50) | 1.02(±0.39) | 1.10(±0.32) |
| hsa-miR-378a-3 | 1.00(±0.28) | 1.13(±0.43) | 1.21(±0.51) | 1.08(±0.35) | hsa-miR-210 | 1.00(±0.04) | 0.74(±0.24) | 0.71(±0.12) | 0.85(±0.23) |
| hsa-let-7b-5p | 1.00(±0.07) | 0.83(±0.03) | 0.46(±0.01) | 0.75(±0.01) | hsa-miR-17-5p | 1.00(±0.23) | 1.03(±0.31) | 0.78(±0.20) | 0.90(±0.11) |
| hsa-miR-205-5p | 1.00(±0.14) | 0.58(±0.24) | 0.67(±0.27) | 0.46(±0.12) | hsa-miR-98-5p | 1.00(±0.11) | 2.01(±0.32) | 0.75(±0.13) | 1.66(±0.14) |
| hsa-miR-181a-5 | 1.00(±0.08) | 0.94(±0.16) | 0.86(±0.14) | 0.60(±0.05) | hsa-miR-34a-5p | 1.00(±0.24) | 1.20(±0.35) | 1.13(±0.52) | 1.24(±0.24) |
| hsa-miR-130a-3 | 1.00(±0.39) | 0.36(±0.43) | 0.32(±0.29) | 0.30(±0.31) | hsa-miR-25-3p | 1.00(±0.41) | 0.82(±0.25) | 0.61(±0.19) | 0.87(±0.17) |
| hsa-miR-140-5p | 1.00(±0.33) | 1.25(±0.69) | 0.98(±0.34) | 1.13(±0.44) | hsa-miR-144-3p | 1.00(±0.07) | 2.32(±3.30) | 0.34(±0.04) | 0.85(±0.30) |
| hsa-miR-20a-5p | 1.00(±0.21) | 1.20(±0.53) | 1.09(±0.44) | 1.20(±0.33) | hsa-miR-128 | 1.00(±0.38) | 1.09(±0.57) | 0.83(±0.54) | 1.05(±0.39) |
| hsa-miR-146b-5 | 1.00(±0.17) | 0.72(±0.20) | 0.55(±0.14) | 0.67(±0.17) | hsa-miR-143-3p | 1.00(±0.07) | 0.56(±0.03) | 0.37(±0.08) | 0.87(±0.02) |
| hsa-miR-132-3p | 1.00(±0.23) | 1.60(±0.50) | 1.02(±0.43) | 1.57(±0.52) | hsa-miR-215 | 1.00(±0.06) | 0.77(±0.18) | 0.38(±0.03) | 0.71(±0.24) |
| hsa-miR-193b-3 | 1.00(±0.01) | 0.93(±0.10) | 0.95(±0.01) | 0.88(±0.06) | hsa-miR-19a-3p | 1.00(±0.43) | 1.11(±0.58) | 0.82(±0.56) | 0.93(±0.28) |
| hsa-miR-183-5p | 1.00(±0.35) | 1.10(±0.51) | 1.05(±0.33) | 1.07(±0.36) | hsa-miR-193a-5 | 1.00(±0.06) | 0.86(±0.08) | 0.68(±0.06) | 0.77(±0.09) |
| hsa-miR-34c-5p | 1.00(±0.41) | 0.44(±0.30) | 0.51(±0.19) | 0.48(±0.16) | hsa-miR-18a-5p | 1.00(±0.47) | 1.23(±0.77) | 0.99(±0.75) | 1.10(±0.52) |
| hsa-miR-30c-5p | 1.00(±0.11) | 1.19(±0.34) | 1.00(±0.24) | 1.04(±0.19) | hsa-miR-125b-5 | 1.00(±0.27) | 0.62(±0.24) | 0.81(±0.37) | 0.51(±0.15) |
| hsa-miR-148a-3 | 1.00(±0.36) | 1.17(±0.48) | 0.88(±0.36) | 1.07(±0.33) | hsa-miR-126-3p | 1.00(±0.22) | 1.00(±0.33) | 0.82(±0.28) | 0.89(±0.17) |
| Hsa0--miR-134 | 1.00(±0.07) | 0.54(±0.09) | 1.04(±0.38) | 0.64(±0.02) | hsa-miR-27a-3p | 1.00(±0.38) | 0.99(±0.48) | 0.79(±0.43) | 0.87(±0.29) |
| hsa-let-7g-5p | 1.00(±0.09) | 1.31(±0.43) | 0.74(±0.21) | 1.09(±0.43) | hsa-miR-372 | 1.00(±0.10) | 0.45(±0.03) | 0.38(±0.06) | 0.34(±0.01) |
| hsa-miR-138-5p | 1.00(±0.04) | 1.01(±0.22) | 0.92(±0.12) | 0.93(±0.12) | hsa-miR-149-5p | 1.00(±0.44) | 1.07(±0.64) | 0.77(±0.46) | 0.95(±0.41) |
| hsa-miR-373-3p | 1.00(±0.28) | 1.02(±0.33) | 1.08(±0.10) | 0.89(±0.29) | hsa-miR-23b-3p | 1.00(±0.43) | 1.04(±0.52) | 0.87(±0.58) | 1.19(±0.42) |
| hsa-let-7c | 1.00(±0.10) | 0.52(±0.12) | 0.51(±0.09) | 0.46(±0.12) | hsa-miR-203a | 1.00(±0.05) | 0.49(±0.02) | 0.47(±0.11) | 0.86(±0.03) |
| hsa-let-7e-5p | 1.00(±0.30) | 0.88(±0.20) | 0.75(±0.11) | 0.89(±0.14) | hsa-miR-32-5p | 1.00(±0.06) | 0.56(±0.09) | 0.57(±0.23) | 1.25(±0.08) |
| hsa-miR-218-5p | 1.00(±0.13) | 1.29(±0.37) | 0.96(±0.24) | 1.23(±0.29) | hsa-miR-181c-5 | 1.00(±0.11) | 0.85(±0.23) | 0.90(±0.24) | 0.71(±0.06) |
| hsa-miR-29b-3p | 1.00(±0.30) | 0.93(±0.36) | 0.88(±0.30) | 0.80(±0.20) | cel-miR-39-3p | 1.00(±0.13) | 0.73(±0.22) | 0.68(±0.26) | 0.62(±0.05) |
| hsa-miR-146a-5 | 1.00(±0.10) | 0.82(±0.06) | 0.84(±0.05) | 0.65(±0.01) | cel-miR-39-3p | 1.00(±0.03) | 0.69(±0.31) | 0.62(±0.10) | 0.77(±0.11) |
| hsa-miR-135b-5 | 1.00(±0.01) | 1.16(±0.13) | 0.67(±0.05) | 1.24(±0.09) | SNORD61 | 1.00(±0.19) | 1.00(±0.41) | 1.00(±0.43) | 1.00(±0.28) |
| hsa-miR-206 | 1.00(±0.26) | 0.79(±0.19) | 0.66(±0.06) | 0.82(±0.10) | SNORD68 | 1.00(±0.47) | 1.13(±0.86) | 1.02(±0.67) | 1.17(±0.59) |
| hsa-miR-124-3p | 1.00(±0.10) | 0.47(±0.04) | 0.82(±0.12) | 0.93(±0.07) | SNORD72 | 1.00(±0.24) | 0.95(±0.44) | 1.02(±0.50) | 0.94(±0.23) |
| hsa-miR-21-5p | 1.00(±0.19) | 1.09(±0.30) | 1.04(±0.32) | 1.07(±0.22) | SNORD95 | 1.00(±0.28) | 1.05(±0.38) | 0.99(±0.32) | 1.17(±0.29) |
| hsa-miR-181d | 1.00(±0.01) | 0.49(±0.05) | 0.56(±0.05) | 0.39(±0.03) | SNORD96A | 1.00(±0.28) | 1.15(±0.54) | 1.01(±0.44) | 1.13(±0.32) |
| hsa-miR-301a-3 | 1.00(±0.49) | 0.92(±0.64) | 1.05(±0.67) | 0.83(±0.28) | RNU6B | 1.00(±18) | 0.90(±0.17) | 0.86(±0.12) | 0.95(±0.06) |
| hsa-miR-200c-3 | 1.00(±0.08) | 0.72(±0.09) | 0.62(±0.03) | 0.59(±0.05) | miRTC | 1.00(±4.24) | 0.92(±4.38) | 0.87(±3.70) | 0.90(±3.44) |
| hsa-miR-100-5p | 1.00(±0.02) | 1.07(±0.11) | 1.01(±0.07) | 1.14(±0.08) | miRTC | 1.00(±5.11) | 0.91(±4.84) | 0.82(±4.01) | 0.90(±3.86) |
| hsa-miR-10b-5p | 1.00(±0.03) | 0.80(±0.05) | 0.67(±0.11) | 0.78(±0.07) | PCC | 1.00(±0.22) | 0.97(±0.42) | 1.04(±0.52) | 1.00(±0.51) |
| hsa-miR-155-5p | 1.00(±0.18) | 0.29(±0.05) | 1.14(±0.14) | 0.89(±0.13) | PCC | 1.00(±0.17) | 0.97(±0.36) | 1.19(±0.42) | 1.04(±0.42) |
Suppl Table 1
