# Supplementary material for: A new KSRP-binding compound suppresses distant metastasis of colorectal cancer by targeting the oncogenic KITENIN complex
Source: Mol Cancer. 2021 May 26;20:78. doi: 10.1186/s12943-021-01368-w (PMC8152081; doi:10.1186/s12943-021-01368-w)
Supplement: Supplementary file 8 — Additional file 8: Supplementary Table 2. Changes in tumor suppressor microRNAs under KITENIN overexpression, and the effects of DKC1125 on these changes. Based on the results of suppressor miRNA in KITENIN-overexpressed CRC cells (Supplementary Table 1), suppressor miRNAs whose levels changed in CRC cells expressing high KITENIN and following treatment with DKC1125 were investigated using the Pathway-focused miScript miRNA PCR array. Data were represented as mean ± SEM (n = 3). [file 12943_2021_1368_MOESM8_ESM.pptx]

## Slide 1
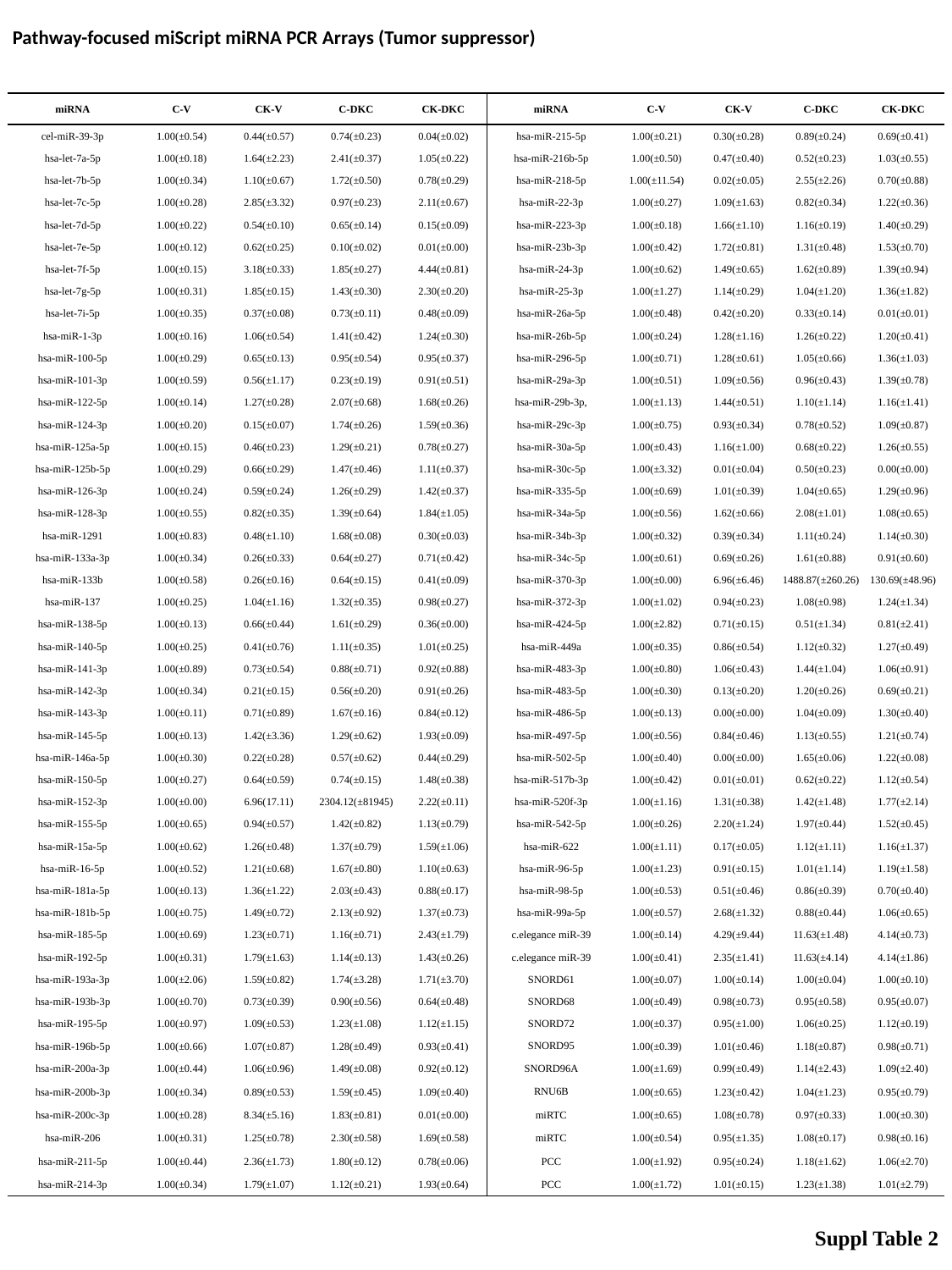

Pathway-focused miScript miRNA PCR Arrays (Tumor suppressor)
| miRNA | C-V | CK-V | C-DKC | CK-DKC | miRNA | C-V | CK-V | C-DKC | CK-DKC |
| --- | --- | --- | --- | --- | --- | --- | --- | --- | --- |
| cel-miR-39-3p | 1.00(±0.54) | 0.44(±0.57) | 0.74(±0.23) | 0.04(±0.02) | hsa-miR-215-5p | 1.00(±0.21) | 0.30(±0.28) | 0.89(±0.24) | 0.69(±0.41) |
| hsa-let-7a-5p | 1.00(±0.18) | 1.64(±2.23) | 2.41(±0.37) | 1.05(±0.22) | hsa-miR-216b-5p | 1.00(±0.50) | 0.47(±0.40) | 0.52(±0.23) | 1.03(±0.55) |
| hsa-let-7b-5p | 1.00(±0.34) | 1.10(±0.67) | 1.72(±0.50) | 0.78(±0.29) | hsa-miR-218-5p | 1.00(±11.54) | 0.02(±0.05) | 2.55(±2.26) | 0.70(±0.88) |
| hsa-let-7c-5p | 1.00(±0.28) | 2.85(±3.32) | 0.97(±0.23) | 2.11(±0.67) | hsa-miR-22-3p | 1.00(±0.27) | 1.09(±1.63) | 0.82(±0.34) | 1.22(±0.36) |
| hsa-let-7d-5p | 1.00(±0.22) | 0.54(±0.10) | 0.65(±0.14) | 0.15(±0.09) | hsa-miR-223-3p | 1.00(±0.18) | 1.66(±1.10) | 1.16(±0.19) | 1.40(±0.29) |
| hsa-let-7e-5p | 1.00(±0.12) | 0.62(±0.25) | 0.10(±0.02) | 0.01(±0.00) | hsa-miR-23b-3p | 1.00(±0.42) | 1.72(±0.81) | 1.31(±0.48) | 1.53(±0.70) |
| hsa-let-7f-5p | 1.00(±0.15) | 3.18(±0.33) | 1.85(±0.27) | 4.44(±0.81) | hsa-miR-24-3p | 1.00(±0.62) | 1.49(±0.65) | 1.62(±0.89) | 1.39(±0.94) |
| hsa-let-7g-5p | 1.00(±0.31) | 1.85(±0.15) | 1.43(±0.30) | 2.30(±0.20) | hsa-miR-25-3p | 1.00(±1.27) | 1.14(±0.29) | 1.04(±1.20) | 1.36(±1.82) |
| hsa-let-7i-5p | 1.00(±0.35) | 0.37(±0.08) | 0.73(±0.11) | 0.48(±0.09) | hsa-miR-26a-5p | 1.00(±0.48) | 0.42(±0.20) | 0.33(±0.14) | 0.01(±0.01) |
| hsa-miR-1-3p | 1.00(±0.16) | 1.06(±0.54) | 1.41(±0.42) | 1.24(±0.30) | hsa-miR-26b-5p | 1.00(±0.24) | 1.28(±1.16) | 1.26(±0.22) | 1.20(±0.41) |
| hsa-miR-100-5p | 1.00(±0.29) | 0.65(±0.13) | 0.95(±0.54) | 0.95(±0.37) | hsa-miR-296-5p | 1.00(±0.71) | 1.28(±0.61) | 1.05(±0.66) | 1.36(±1.03) |
| hsa-miR-101-3p | 1.00(±0.59) | 0.56(±1.17) | 0.23(±0.19) | 0.91(±0.51) | hsa-miR-29a-3p | 1.00(±0.51) | 1.09(±0.56) | 0.96(±0.43) | 1.39(±0.78) |
| hsa-miR-122-5p | 1.00(±0.14) | 1.27(±0.28) | 2.07(±0.68) | 1.68(±0.26) | hsa-miR-29b-3p, | 1.00(±1.13) | 1.44(±0.51) | 1.10(±1.14) | 1.16(±1.41) |
| hsa-miR-124-3p | 1.00(±0.20) | 0.15(±0.07) | 1.74(±0.26) | 1.59(±0.36) | hsa-miR-29c-3p | 1.00(±0.75) | 0.93(±0.34) | 0.78(±0.52) | 1.09(±0.87) |
| hsa-miR-125a-5p | 1.00(±0.15) | 0.46(±0.23) | 1.29(±0.21) | 0.78(±0.27) | hsa-miR-30a-5p | 1.00(±0.43) | 1.16(±1.00) | 0.68(±0.22) | 1.26(±0.55) |
| hsa-miR-125b-5p | 1.00(±0.29) | 0.66(±0.29) | 1.47(±0.46) | 1.11(±0.37) | hsa-miR-30c-5p | 1.00(±3.32) | 0.01(±0.04) | 0.50(±0.23) | 0.00(±0.00) |
| hsa-miR-126-3p | 1.00(±0.24) | 0.59(±0.24) | 1.26(±0.29) | 1.42(±0.37) | hsa-miR-335-5p | 1.00(±0.69) | 1.01(±0.39) | 1.04(±0.65) | 1.29(±0.96) |
| hsa-miR-128-3p | 1.00(±0.55) | 0.82(±0.35) | 1.39(±0.64) | 1.84(±1.05) | hsa-miR-34a-5p | 1.00(±0.56) | 1.62(±0.66) | 2.08(±1.01) | 1.08(±0.65) |
| hsa-miR-1291 | 1.00(±0.83) | 0.48(±1.10) | 1.68(±0.08) | 0.30(±0.03) | hsa-miR-34b-3p | 1.00(±0.32) | 0.39(±0.34) | 1.11(±0.24) | 1.14(±0.30) |
| hsa-miR-133a-3p | 1.00(±0.34) | 0.26(±0.33) | 0.64(±0.27) | 0.71(±0.42) | hsa-miR-34c-5p | 1.00(±0.61) | 0.69(±0.26) | 1.61(±0.88) | 0.91(±0.60) |
| hsa-miR-133b | 1.00(±0.58) | 0.26(±0.16) | 0.64(±0.15) | 0.41(±0.09) | hsa-miR-370-3p | 1.00(±0.00) | 6.96(±6.46) | 1488.87(±260.26) | 130.69(±48.96) |
| hsa-miR-137 | 1.00(±0.25) | 1.04(±1.16) | 1.32(±0.35) | 0.98(±0.27) | hsa-miR-372-3p | 1.00(±1.02) | 0.94(±0.23) | 1.08(±0.98) | 1.24(±1.34) |
| hsa-miR-138-5p | 1.00(±0.13) | 0.66(±0.44) | 1.61(±0.29) | 0.36(±0.00) | hsa-miR-424-5p | 1.00(±2.82) | 0.71(±0.15) | 0.51(±1.34) | 0.81(±2.41) |
| hsa-miR-140-5p | 1.00(±0.25) | 0.41(±0.76) | 1.11(±0.35) | 1.01(±0.25) | hsa-miR-449a | 1.00(±0.35) | 0.86(±0.54) | 1.12(±0.32) | 1.27(±0.49) |
| hsa-miR-141-3p | 1.00(±0.89) | 0.73(±0.54) | 0.88(±0.71) | 0.92(±0.88) | hsa-miR-483-3p | 1.00(±0.80) | 1.06(±0.43) | 1.44(±1.04) | 1.06(±0.91) |
| hsa-miR-142-3p | 1.00(±0.34) | 0.21(±0.15) | 0.56(±0.20) | 0.91(±0.26) | hsa-miR-483-5p | 1.00(±0.30) | 0.13(±0.20) | 1.20(±0.26) | 0.69(±0.21) |
| hsa-miR-143-3p | 1.00(±0.11) | 0.71(±0.89) | 1.67(±0.16) | 0.84(±0.12) | hsa-miR-486-5p | 1.00(±0.13) | 0.00(±0.00) | 1.04(±0.09) | 1.30(±0.40) |
| hsa-miR-145-5p | 1.00(±0.13) | 1.42(±3.36) | 1.29(±0.62) | 1.93(±0.09) | hsa-miR-497-5p | 1.00(±0.56) | 0.84(±0.46) | 1.13(±0.55) | 1.21(±0.74) |
| hsa-miR-146a-5p | 1.00(±0.30) | 0.22(±0.28) | 0.57(±0.62) | 0.44(±0.29) | hsa-miR-502-5p | 1.00(±0.40) | 0.00(±0.00) | 1.65(±0.06) | 1.22(±0.08) |
| hsa-miR-150-5p | 1.00(±0.27) | 0.64(±0.59) | 0.74(±0.15) | 1.48(±0.38) | hsa-miR-517b-3p | 1.00(±0.42) | 0.01(±0.01) | 0.62(±0.22) | 1.12(±0.54) |
| hsa-miR-152-3p | 1.00(±0.00) | 6.96(17.11) | 2304.12(±81945) | 2.22(±0.11) | hsa-miR-520f-3p | 1.00(±1.16) | 1.31(±0.38) | 1.42(±1.48) | 1.77(±2.14) |
| hsa-miR-155-5p | 1.00(±0.65) | 0.94(±0.57) | 1.42(±0.82) | 1.13(±0.79) | hsa-miR-542-5p | 1.00(±0.26) | 2.20(±1.24) | 1.97(±0.44) | 1.52(±0.45) |
| hsa-miR-15a-5p | 1.00(±0.62) | 1.26(±0.48) | 1.37(±0.79) | 1.59(±1.06) | hsa-miR-622 | 1.00(±1.11) | 0.17(±0.05) | 1.12(±1.11) | 1.16(±1.37) |
| hsa-miR-16-5p | 1.00(±0.52) | 1.21(±0.68) | 1.67(±0.80) | 1.10(±0.63) | hsa-miR-96-5p | 1.00(±1.23) | 0.91(±0.15) | 1.01(±1.14) | 1.19(±1.58) |
| hsa-miR-181a-5p | 1.00(±0.13) | 1.36(±1.22) | 2.03(±0.43) | 0.88(±0.17) | hsa-miR-98-5p | 1.00(±0.53) | 0.51(±0.46) | 0.86(±0.39) | 0.70(±0.40) |
| hsa-miR-181b-5p | 1.00(±0.75) | 1.49(±0.72) | 2.13(±0.92) | 1.37(±0.73) | hsa-miR-99a-5p | 1.00(±0.57) | 2.68(±1.32) | 0.88(±0.44) | 1.06(±0.65) |
| hsa-miR-185-5p | 1.00(±0.69) | 1.23(±0.71) | 1.16(±0.71) | 2.43(±1.79) | c.elegance miR-39 | 1.00(±0.14) | 4.29(±9.44) | 11.63(±1.48) | 4.14(±0.73) |
| hsa-miR-192-5p | 1.00(±0.31) | 1.79(±1.63) | 1.14(±0.13) | 1.43(±0.26) | c.elegance miR-39 | 1.00(±0.41) | 2.35(±1.41) | 11.63(±4.14) | 4.14(±1.86) |
| hsa-miR-193a-3p | 1.00(±2.06) | 1.59(±0.82) | 1.74(±3.28) | 1.71(±3.70) | SNORD61 | 1.00(±0.07) | 1.00(±0.14) | 1.00(±0.04) | 1.00(±0.10) |
| hsa-miR-193b-3p | 1.00(±0.70) | 0.73(±0.39) | 0.90(±0.56) | 0.64(±0.48) | SNORD68 | 1.00(±0.49) | 0.98(±0.73) | 0.95(±0.58) | 0.95(±0.07) |
| hsa-miR-195-5p | 1.00(±0.97) | 1.09(±0.53) | 1.23(±1.08) | 1.12(±1.15) | SNORD72 | 1.00(±0.37) | 0.95(±1.00) | 1.06(±0.25) | 1.12(±0.19) |
| hsa-miR-196b-5p | 1.00(±0.66) | 1.07(±0.87) | 1.28(±0.49) | 0.93(±0.41) | SNORD95 | 1.00(±0.39) | 1.01(±0.46) | 1.18(±0.87) | 0.98(±0.71) |
| hsa-miR-200a-3p | 1.00(±0.44) | 1.06(±0.96) | 1.49(±0.08) | 0.92(±0.12) | SNORD96A | 1.00(±1.69) | 0.99(±0.49) | 1.14(±2.43) | 1.09(±2.40) |
| hsa-miR-200b-3p | 1.00(±0.34) | 0.89(±0.53) | 1.59(±0.45) | 1.09(±0.40) | RNU6B | 1.00(±0.65) | 1.23(±0.42) | 1.04(±1.23) | 0.95(±0.79) |
| hsa-miR-200c-3p | 1.00(±0.28) | 8.34(±5.16) | 1.83(±0.81) | 0.01(±0.00) | miRTC | 1.00(±0.65) | 1.08(±0.78) | 0.97(±0.33) | 1.00(±0.30) |
| hsa-miR-206 | 1.00(±0.31) | 1.25(±0.78) | 2.30(±0.58) | 1.69(±0.58) | miRTC | 1.00(±0.54) | 0.95(±1.35) | 1.08(±0.17) | 0.98(±0.16) |
| hsa-miR-211-5p | 1.00(±0.44) | 2.36(±1.73) | 1.80(±0.12) | 0.78(±0.06) | PCC | 1.00(±1.92) | 0.95(±0.24) | 1.18(±1.62) | 1.06(±2.70) |
| hsa-miR-214-3p | 1.00(±0.34) | 1.79(±1.07) | 1.12(±0.21) | 1.93(±0.64) | PCC | 1.00(±1.72) | 1.01(±0.15) | 1.23(±1.38) | 1.01(±2.79) |
Suppl Table 2
