# Supplementary material for: A new KSRP-binding compound suppresses distant metastasis of colorectal cancer by targeting the oncogenic KITENIN complex
Source: Mol Cancer. 2021 May 26;20:78. doi: 10.1186/s12943-021-01368-w (PMC8152081; doi:10.1186/s12943-021-01368-w)
Supplement: Supplementary file 9 — Additional file 9: Supplementary Table 3. Several suppressor microRNAs whose levels decreased under KITENIN overexpression but recovered after DKC1125. Suppressor miRNAs whose levels were decreased to a greater extent in CRC cells expressing high KITENIN than in parental cells (> 25%) and restored after treatment with DKC1125 (Supplementary Table 1 and 2) are summarized. Data were represented as mean ± SEM (n = 3). In addition to miR-124-3p, miR-143-3p, and miR-150-5p, several other suppressor miRNAs are shown: miR-100-5p, miR-125a-5p, miR-125b-5p, miR-140-5p, miR-218-5p, miR-34c-5p, miR-98-5p, miR-101-3p, miR-133a-3p, miR-216b-5p, miR-34b-3p, miR-486-5p, miR-502-5p, and miR-622. [file 12943_2021_1368_MOESM9_ESM.pptx]

## Slide 1
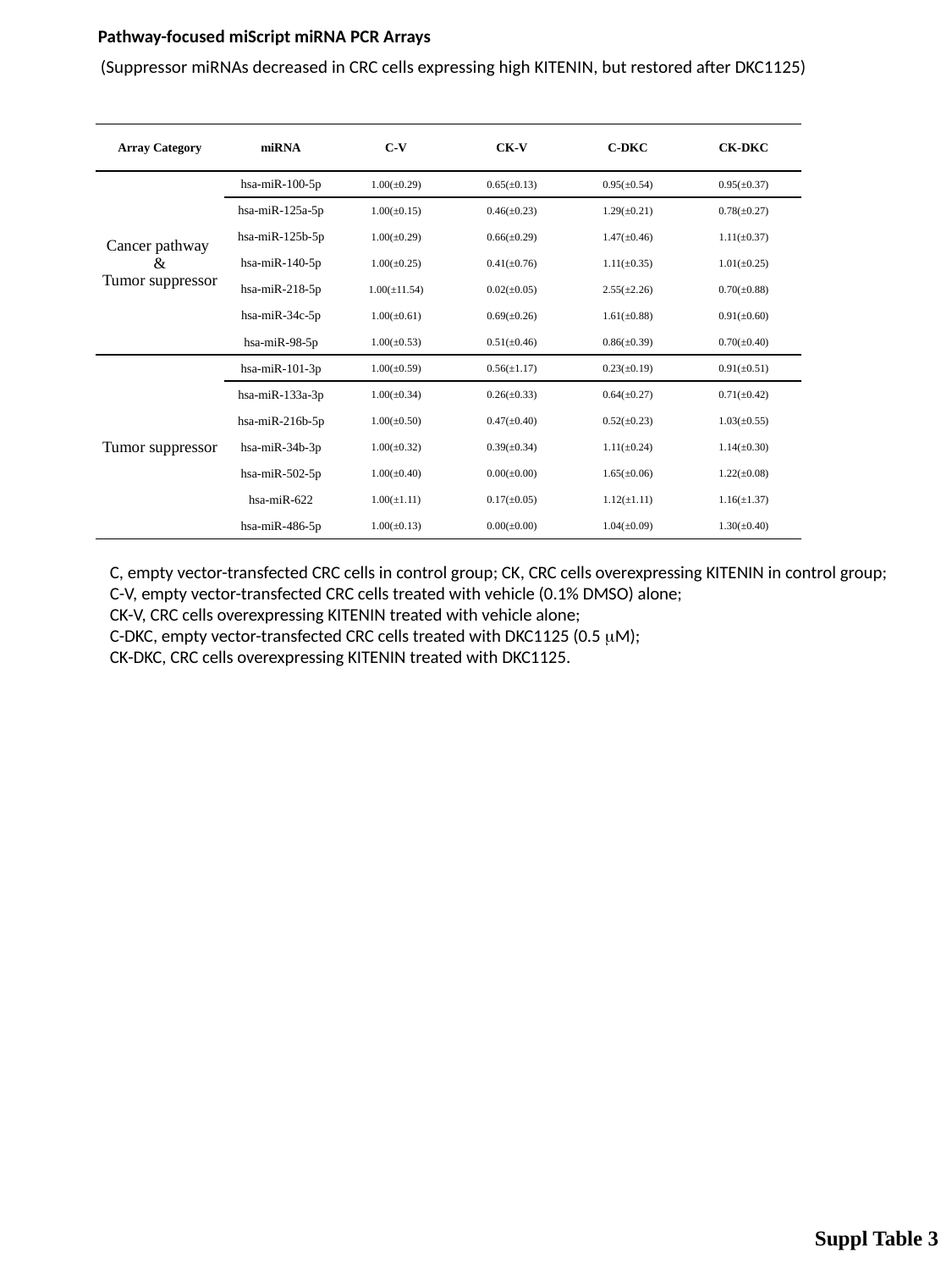

Pathway-focused miScript miRNA PCR Arrays
(Suppressor miRNAs decreased in CRC cells expressing high KITENIN, but restored after DKC1125)
| Array Category | miRNA | C-V | CK-V | C-DKC | CK-DKC |
| --- | --- | --- | --- | --- | --- |
| Cancer pathway & Tumor suppressor | hsa-miR-100-5p | 1.00(±0.29) | 0.65(±0.13) | 0.95(±0.54) | 0.95(±0.37) |
| | hsa-miR-125a-5p | 1.00(±0.15) | 0.46(±0.23) | 1.29(±0.21) | 0.78(±0.27) |
| | hsa-miR-125b-5p | 1.00(±0.29) | 0.66(±0.29) | 1.47(±0.46) | 1.11(±0.37) |
| | hsa-miR-140-5p | 1.00(±0.25) | 0.41(±0.76) | 1.11(±0.35) | 1.01(±0.25) |
| | hsa-miR-218-5p | 1.00(±11.54) | 0.02(±0.05) | 2.55(±2.26) | 0.70(±0.88) |
| | hsa-miR-34c-5p | 1.00(±0.61) | 0.69(±0.26) | 1.61(±0.88) | 0.91(±0.60) |
| | hsa-miR-98-5p | 1.00(±0.53) | 0.51(±0.46) | 0.86(±0.39) | 0.70(±0.40) |
| Tumor suppressor | hsa-miR-101-3p | 1.00(±0.59) | 0.56(±1.17) | 0.23(±0.19) | 0.91(±0.51) |
| | hsa-miR-133a-3p | 1.00(±0.34) | 0.26(±0.33) | 0.64(±0.27) | 0.71(±0.42) |
| | hsa-miR-216b-5p | 1.00(±0.50) | 0.47(±0.40) | 0.52(±0.23) | 1.03(±0.55) |
| | hsa-miR-34b-3p | 1.00(±0.32) | 0.39(±0.34) | 1.11(±0.24) | 1.14(±0.30) |
| | hsa-miR-502-5p | 1.00(±0.40) | 0.00(±0.00) | 1.65(±0.06) | 1.22(±0.08) |
| | hsa-miR-622 | 1.00(±1.11) | 0.17(±0.05) | 1.12(±1.11) | 1.16(±1.37) |
| | hsa-miR-486-5p | 1.00(±0.13) | 0.00(±0.00) | 1.04(±0.09) | 1.30(±0.40) |
C, empty vector-transfected CRC cells in control group; CK, CRC cells overexpressing KITENIN in control group;
C-V, empty vector-transfected CRC cells treated with vehicle (0.1% DMSO) alone;
CK-V, CRC cells overexpressing KITENIN treated with vehicle alone;
C-DKC, empty vector-transfected CRC cells treated with DKC1125 (0.5 μM);
CK-DKC, CRC cells overexpressing KITENIN treated with DKC1125.
Suppl Table 3
